# Regulation of *CLB6* expression by the cytoplasmic deadenylase Ccr4 through its coding and 3’ UTR regions

**DOI:** 10.1371/journal.pone.0268283

**Published:** 2022-05-06

**Authors:** Jastin Edrian Cocuangco Revilleza, Megumi Sato, Kaoru Irie, Yasuyuki Suda, Tomoaki Mizuno, Kenji Irie

**Affiliations:** 1 Faculty of Medicine, Department of Molecular Cell Biology, University of Tsukuba, Tsukuba, Japan; 2 Doctoral Program in Biomedical Sciences, Graduate School of Comprehensive Human Sciences, University of Tsukuba, Tsukuba, Japan; 3 Live Cell Super-resolution Imaging Research Team, RIKEN Center for Advanced Photonics, Wako, Saitama, Japan; Kindai University: Kinki Daigaku, JAPAN

## Abstract

RNA stability control contributes to the proper expression of gene products. Messenger RNAs (mRNAs) in eukaryotic cells possess a 5’ cap structure and the 3’ poly(A) tail which are important for mRNA stability and efficient translation. The Ccr4-Not complex is a major cytoplasmic deadenylase and functions in mRNA degradation. The *CLB1-6* genes in *Saccharomyces cerevisiae* encode B-type cyclins which are involved in the cell cycle progression together with the cyclin-dependent kinase Cdc28. The *CLB* genes consist of *CLB1/2*, *CLB3/4*, and *CLB5/6* whose gene products accumulate at the G2-M, S-G2, and late G1 phase, respectively. These Clb protein levels are thought to be mainly regulated by the transcriptional control and the protein stability control. Here we investigated regulation of *CLB1-6* expression by Ccr4. Our results show that all *CLB1-6* mRNA levels were significantly increased in the *ccr4Δ* mutant compared to those in wild-type cells. Clb1, Clb4, and Clb6 protein levels were slightly increased in the *ccr4Δ* mutant, but the Clb2, Clb3, and Clb5 protein levels were similar to those in wild-type cells. Since both *CLB6* mRNA and Clb6 protein levels were most significantly increased in the *ccr4Δ* mutant, we further analyzed the *cis*-elements for the Ccr4-mediated regulation within *CLB6* mRNA. We found that there were destabilizing sequences in both coding sequence and 3’ untranslated region (3’ UTR). The destabilizing sequences in the coding region were found to be both within and outside the sequences corresponding the cyclin domain. The *CLB6* 3’ UTR was sufficient for mRNA destabilization and decrease of the reporter GFP gene and this destabilization involved Ccr4. Our results suggest that *CLB6* expression is regulated by Ccr4 through the coding sequence and 3’ UTR of *CLB6* mRNA.

## Introduction

The cells contain the same DNA, but each cell contains a different subset of expressed genes. There are different points at which cells determine which genes are copied into RNA and translated to proteins [1]. Once the mRNA has been made, it gradually moves to the ribosome where it is translated and the regulation via RNA-binding proteins and small regulatory RNA molecules is a common mechanism for developmental control [1]. Eukaryotic pre-mRNAs are initially modified at the 5*’* and 3*’* ends. At the 5’ end, several enzymes synthesize the 5’ cap, a 7-methylguanylate that protects an mRNA from enzymatic degradation and assist in cytoplasm transport. At the 3*’* end, it is cleaved by an endonuclease to yield a free 3’-hydroxyl group wherein a sequence of adenylic acid residues is added by a poly(A) polymerase [1]. Gene expression can be regulated at many steps in the pathway from DNA to protein, one of which is selectively destabilizing certain mRNA molecules in the cytoplasm or called mRNA degradation control [1]. mRNA degradation is modulated by small RNA molecules or RNA-binding proteins [1,2].

RNases are responsible for mediating the processing, decay, and quality control of RNA [3]. The stability of mRNA largely depends on the mRNA sequence, which affects the accessibility of various RNA-binding proteins or small RNAs to the mRNAs [4]. Most mRNAs including the AU-rich element (ARE)-containing mRNAs involves 3’ untranslated region (UTR) destabilizing elements [5]. Most mRNAs undergo a deadenylation-dependent decay where the poly(A) tail is removed by a deadenylase activity of the Ccr4-Not complex [6]. The deadenylated mRNAs are further degraded by decapping enzymes and 5’-3’ exonuclease, or 3’-5’ exonucleases.

The Ccr4-Not complex is the main deadenylase in all eukaryotes including the budding yeast, *Saccharomyces cerevisiae* [5–8]. This is a multi-subunit protein complex which has a big contribution to regulate RNA metabolism from synthesis to decay [8]. Several studies have shown its role in mRNA decay in *S*. *cerevisiae*. First, Pumilio-homology domain Family (Puf) protein, which binds to the 3’ UTR of target mRNA, recruits the Ccr4-Not complex to the target mRNA and stimulates deadenylation [9]. Next, the Ccr4-Not complex contributes to septin organization via the deadenylation of the mRNAs encoding the septin regulators [10]. Our lab also reported the role of Ccr4 in the regulation of *LRG1* mRNA in which Ccr4 regulates not only the mRNA level through poly(A) shortening, but also its translation [11]. Recently, we also reported that the polyA-binding protein (Pab1)-binding protein, Pbp1, mediates the growth defect caused by the deletion of *ccr4Δ* and *pop2Δ* [12]. Indeed, the Ccr4-Not complex is a global regulator of gene expression from yeast to human [8,13].

The cell cycle progression is regulated by cyclin-dependent protein kinases (Cdk) [1]. Cdk1 is present at a constant level during the cell cycle. Cyclin protein levels vary in concentrations and act as a signal for the transition between phases [1]. In *S*. *cerevisiae*, 6 Cyclin B genes (*CLB*) are classified into 3 types with different stages of accumulation: *CLB1/CLB2 CLB3/CLB4*, and *CLB5/CLB6* accumulate during the G2 and M, S and G2 and late G1 phase, respectively [1,14,15]. Cyclin degradation is essential for the cell cycle progression [1]. It was previously reported that Cyclin B is degraded by the ubiquitin pathway which is the most selective degradation pattern in eukaryotic cells [1,16] and the proteolysis of cyclin potentially plays a role in proper cell cycle progression [17–19]. However, this claim is still unclear or unknown whether mRNA stability regulation is involved in Clb protein level. It was previously reported that *CLB* mRNA accumulation is dependent on the heterogeneous nuclear ribonucleoprotein arginine methyltransferase (Hmt1) [20]. Another study also showed that mRNA stability of *CLB2* is controlled in its promoter-dependent manner [21]. Lastly, it was also demonstrated that the endoribonuclease MRP cleaves the *CLB2* mRNA in its 5’ UTR for rapid 5’ to 3’ degradation by the Xrn1 nuclease [22].

In this study, we investigated the role of Ccr4 on the expression of *CLB1-6* mRNAs. By creating multiple gene cassettes of *CLB1-6* genes, we were able to determine which region is responsible for its recognition by Ccr4 for degradation. Our results show that *CLB6* expression is regulated by Ccr4 through the coding sequence and 3’ UTR of *CLB6* mRNA.

## Materials and methods

### Strains and media

*Escherichia coli* DH5α strain was used for DNA manipulations. The yeast strains used in this study are isogenic derivatives of the W303 strain and listed in S1 Table. Gene deletions were conducted to replace the target gene with resistance cassettes by homologous recombination using standard PCR-based method [23]. Colony PCR was conducted with forming clones to confirm complete deletion at the expected locus. The media used in this study included YPD (1% yeast extract, 2% peptone, and 2% glucose) and SC (synthetic minimal medium). SC media lacking amino acids or other nutrients (e.g. SC-ura corresponding to SC lacking uracil) were used to select the transformants. General procedures were performed as described previously [24].

### Plasmids

Plasmids used in this study are listed in S2 Table. pCgLEU2, pCgHIS3, and pCgTRP1 are pUC19 carrying the *Candida glabrata LEU2*, *HIS3*, and *TRP1* genes, respectively [25].

Plasmids YCplac33-CLBx-HA-CLBx 3’ UTR, YCplac33-CLBx-HA-ADH1 3’ UTR, and YCplac33-MCM2 promoter-GFP-CLBx 3’ UTR were created using standard PCR-based method previously described [26]. Primers used for plasmid construction are listed in S3, S4, and S5 Tables. The process of creating the 3xHA tag gene construct was done in two sequential steps. First, the *CLBx-HA-ADH1 3’ UTR* was constructed by using a pFA6-3xHA-kanMX6 plasmid (Addgene plasmid # 39292). Next, the *CLBx-HA-CLB 3’ UTR* is constructed by using the gene template from the first step where primers are prepared by pairing the 5’ UTR primers with the *HA-CLBx 3’ UTR* region after the stop codon.

Deletions of *CLB6* coding sequence and 3’ UTR were prepared by using the gene constructs of C*LB6-HA-CLB6 3’ UTR* and *MCM2 promoter-GFP-CLB6 3’ UTR* for the coding region and 3’ UTR, respectively. Primers used for plasmid construction are listed in S6 and S7 Tables. Starting from the second base pair, 50 amino acids were deleted per sequence for the coding region, overlapping the middle 25^th^ for the consecutive number (e.g. deletion 1 is from amino acid 2 to 50 while deletion 2 is from amino acid 25 to 75) with a total of 15 deletions. For the 3’ UTR, 30 bases were deleted per sequence, overlapping the middle 5^th^ for the consecutive number (e.g. deletion 1 is from 2 to 12 while deletion 2 is from 7 to 17) with a total of 6 deletions.

### Cell sampling, sample preparation, and RNA extraction

Yeast cells without plasmids were grown overnight at 28°C using YPD medium. It is then inoculated into 30 ml fresh YPD medium to 0.5 OD. Cell samples of OD_10_ were collected after 4 hours. Yeast cells harboring plasmids were grown overnight at 28°C using SC-ura medium. It is then inoculated into 30 ml fresh SC-ura medium to 0.5 OD. Cell samples of OD_10_ were collected after 4 hours. The cells are washed, spun down and is ready for sample preparation.

The RNA isolation procedure are as follows. After washing, the cells were immersed using ISOGEN reagent (Nippon Gene, Toyama, Japan). It was then mashed using a Micromash MS-100R (Tomy, Japan) for 2x. Chloroform is added, mixed and centrifuged. The aqueous top layer was collected (with the RNA sample) and was precipitated with Isopropanol. The RNA samples were placed at 4°C overnight. On the next day, the cDNAs were prepared using a Primescript RT reagent Kit with gDNA Eraser (Takara, Japan). The cDNAs are finally ready for qRT-PCR analysis.

### qRT-PCR analysis and microarray data

For qRT-PCR, the QuantStudio 5 real-time RT-PCR systems (Applied Biosystems, MA, USA) with SYBR Premix Ex Taq (Takara, Shiga, Japan) was used. The mRNA fold changes in mRNA level were computed using the 2^-ΔΔCt^ method and normalized against the *ACT1* reference gene. Primers are listed on S8 Table. The microarray analysis was performed by the KURABO Bio-Medical Department (Osaka, Japan) using the Agillent-016322 Yeast (V2) Gene Expression 8X15K Microarray. Microarray data sets are available at the Gene Expression Omnibus at http://www.ncbi.nlm.nih.gov/geo (GEO accession number GSE198743).

### Western blot analysis

Yeast cells were cultivated first overnight (16-hours), then a solution having OD_600_ = 0.5 was prepared. For every sampling time, 10 OD of cells was collected from the cultured liquid media to be used for protein extraction. The cells (OD_600_ = 10) of the collected cells were treated with sodium hydroxide for protein extraction [27]. Protein samples were loaded onto an 8% SDS-PAGE gel for protein electrophoresis and then transferred to a PDVF membrane (Merck Millipore, Molsheim, France) for Western blot analysis. The Biomolecular imager LAS-4000 (Fuji Film, Tokyo, Japan) and ODYSSEY CLx (LI-COR, Japan) were used to capture the samples Protein levels were quantified by immunoblotting with anti-HA, anti-GFP and anti-Pgk1 antibodies. The loading control used for the entire study was Pgk1. The protein fold changes in protein level were measured by normalizing with the wild-type.

### Cell cycle synchronization by α-factor block and release

For synchronizing cell cycle, we performed the pheromone-induced cell cycle synchronization procedure previously reported [28]. *MAT***a** *bar1*Δ strains were used to prevent degradation of α-factor. After the overnight culture in YPD medium at 28°C, yeast strains were transferred into fresh YPD medium, and cultured until mid-exponential phase. Then, α-factor was added into the medium and strains were incubated for 2 hours. After the 2-hour exposure to α-factor, 0-minute sample was collected and cells remaining were washed with fresh YPD medium by centrifuge and released by transferring into fresh YPD medium and incubated at 28°C. After start releasing, samples were collected by centrifuge every 10 minutes to 120 minutes.

### Determination of half-lives of *CLB* mRNAs

The half-lives (t_1/2_) of *CLB* mRNAs were investigated by thiolutin-induced inhibition of transcription. We followed the procedure previously reported [29,30]. The *bar1*Δ and the *bar1*Δ *ccr4*Δ mutant cells were pre-cultured in YPD medium at 28°C overnight, and transferred into fresh YPD medium, and cultured until mid-exponential phase. Then, thiolutin (2 mg/ml dissolved in DMSO) was added, and samples were collected by centrifuge at 0, 1, 2, 5, 10, 20, 40, 80 minutes after exposure to thiolutin. The mRNA levels at each time point were determined by qRT-PCR. The half-lives (t_1/2_) were calculated using Microsoft Excel.

## Results

### The *ccr4Δ* mutation showed a synthetic growth defect with the *mih1Δ* mutation

**Fig 1:**
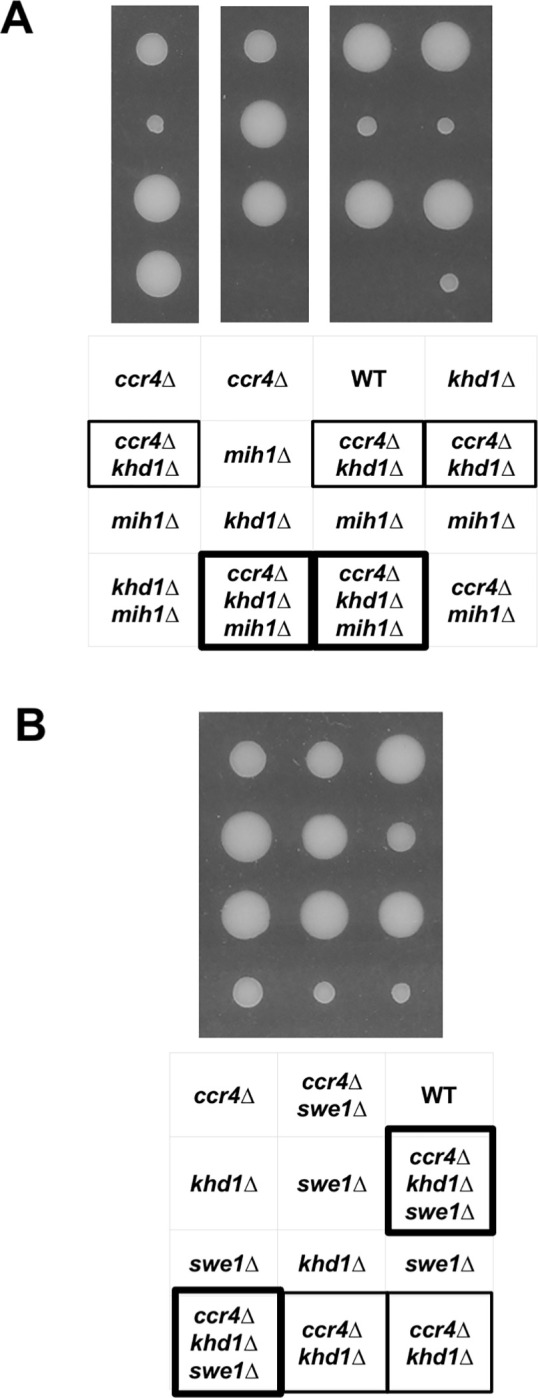
The *ccr4Δ* mutation showed a synthetic growth defect with the *mih1Δ* mutation. The strains that were heterozygous for favorite alleles were sporulated, and tetrads were dissected onto YPD plate. The growth of segregants after 4 days at 30°C is shown. Genotypes are indicated on both sides. More than 50 tetrads were dissected, and representative data are shown. (A) Tetrad analysis of strain 10BD-c4k1m1 that was heterozygous for *ccr4*Δ, *khd1Δ*, and *mih1*Δ alleles. The *ccr4Δ khd1Δ* double mutant strain showed slower growth than the *ccr4*Δ single mutant, and the *ccr4Δ khd1Δ mih1Δ* triple mutant strain was never germinated. (B) Tetrad analysis of strain 10BD-c4k1s1 that was heterozygous for *ccr4*Δ, *khd1Δ*, and *swe1*Δ alleles. The *ccr4Δ khd1Δ swe1Δ* triple mutant showed better growth than the *ccr4Δ khd1Δ* double mutant strain.

It has been suggested that Ccr4 is involved in the cell cycle progression [10,31]. To investigate a possible involvement of Ccr4 in the cell cycle progression, we first examined genetic interactions between *CCR4* and the genes involved in G2-M transition, *MIH1* (mitotic inducer homolog 1), and *SWE1* (Saccharomyces wee1) [32,33]. Since the growth defect of the *ccr4Δ* single mutant strain is difficult to detect, we used the *ccr4Δ khd1Δ* double mutant strain [34,35] (Fig 1). Tetrad analysis revealed that the *mih1*Δ mutant had no obvious phenotype at room temperature, while the *ccr4*Δ mutant grew slowly. We found that the *ccr4*Δ *mih1*Δ double mutant showed slower growth than the *ccr4*Δ single mutant (Fig 1A). The *ccr4Δ khd1Δ* double mutant strain also showed slower growth than the *ccr4*Δ single mutant, and the *ccr4Δ khd1Δ mih1Δ* triple mutant strain was never germinated (Fig 1A). On the other hand, the *ccr4Δ khd1Δ swe1Δ* triple mutant showed better growth than the *ccr4Δ khd1Δ* double mutant strain (Fig 1B). The *ccr4*Δ *swe1*Δ double mutant showed a similar growth with the *ccr4*Δ single mutant (Fig 1B). These results suggest that Ccr4 may have a role in G2-M progression together with Mih1.

### The *CLB1-6* mRNA levels were significantly increased in the *ccr4Δ* mutant

**Fig 2:**
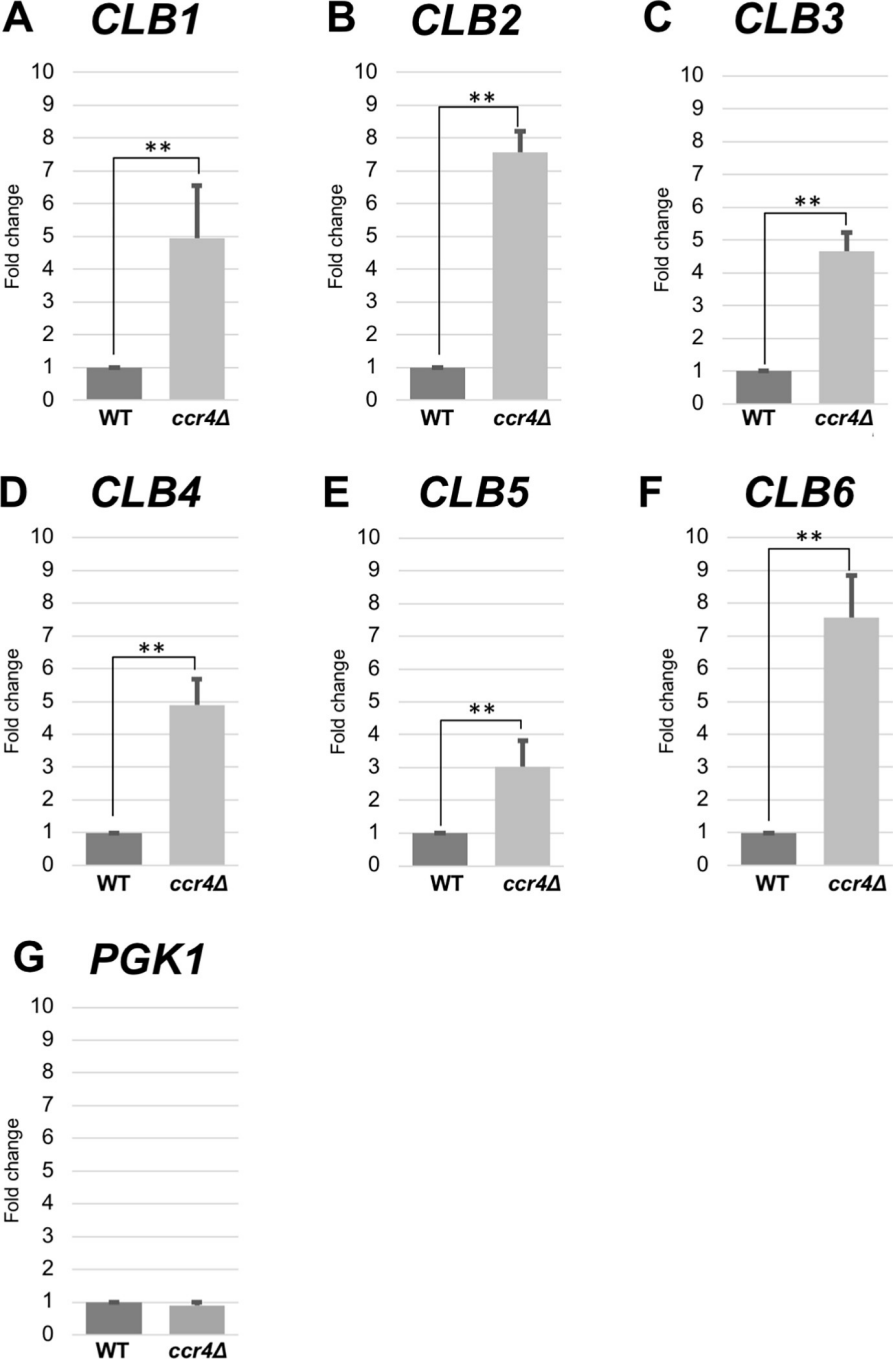
Expression of *CLB1*, *CLB2*, *CLB3*, *CLB4*, *CLB5*, and *CLB6* in wild-type and *ccr4Δ* mutant cells. The mRNA levels of *CLB1* (A), *CLB2* (B), *CLB3* (C), *CLB4* (D), *CLB5* (E), and *CLB6* (F) in *ccr4*Δ mutant strain growing in YPD media relative to the wild-type strain. *PGK1* (G) was used as a control. mRNA levels were quantified by qRT-PCR analysis, and the relative mRNA levels were calculated using 2^-ΔΔCt^ method normalized to *ACT1* reference gene. The data show mean ± SEM (n = 3) of fold change of mRNA level from wild-type cells at 4 h of culture in YPD. *P < 0.05, **P < 0.01 as determined by Tukey’s test.

To investigate a possible role of Ccr4 in G2-M progression, we examined endogenous mRNA levels of *CLB1-6* in wild-type and the *ccr4Δ* mutant cells. All *CLB1-6* mRNA levels were increased in the *ccr4*Δ mutant compared to those in wild-type cells, but its ratio differed in the genes (Fig 2, Table 1). The *CLB2* and *CLB6* mRNA levels were increased about 7.5-fold; the *CLB1*, *CLB3*, and *CLB4* mRNA levels were increased about 5-fold; the *CLB5* mRNA level was increased 3-fold (Fig 2, Table 1). In contrast, the *PGK1* mRNA level was similar in wild-type and the *ccr4*Δ mutant cells. Our previous microarray analyses [12] also support our current results (Table 1): *CLB1*, *CLB2*, *CLB4*, and *CLB6* mRNA levels are increased in the *ccr4Δ* mutant compared to wild-type, while *CLB3* and *CLB5* mRNA levels are not (Table 2). The mRNA levels of G1 cyclin genes, *CLN1-3*, are not increased in the *ccr4Δ* mutant (Table 2). Thus, Ccr4 contributes to the levels of *CLB* mRNAs, but not those of *CLN* mRNAs.

**Table 1:** Summary of endogenous *CLB* mRNA levels in various mutant. All values written are standardized to their respective wild-type values.

	*ccr4Δ*	*pop2Δ*	*not1* (*cdc39*)	*not2* (*cdc36*)	*not4Δ*	*whi3Δ*	*caf20Δ*	*eap1Δ*	*caf20Δ eap1Δ*
*CLB1*	4.69 ± 1.62	3.72 ± 0.25	1.44 ± 0.84	1.54 ± 0.90	2.05 ± 0.23	1.59 ± 0.27	0.88 ± 0.15	0.88 ± 0.17	1.04 ± 0.16
*CLB2*	7.68 ± 0.67	4.12 ± 0.04	1.01 ± 0.58	0.86 ± 0.53	1.76 ± 0.08	1.68 ± 0.24	1.10 ± 0.06	1.10 ± 0.08	1.18 ± 0.07
*CLB3*	4.66 ± 0.56	2.46 ± 0.20	0.88 ± 0.55	0.73 ± 0.42	1.45 ± 0.16	1.75 ± 0.20	0.79 ± 0.10	0.85 ± 0.11	0.78 ± 0.05
*CLB4*	4.89 ± 0.78	3.36 ± 0.29	1.14 ± 0.70	0.73 ± 0.44	2.57 ± 0.16	1.68 ± 0.14	0.91 ± 0.08	0.87 ± 0.02	0.86 ± 0.10
*CLB5*	3.02 ± 0.81	4.83 ± 0.37	1.45 ± 0.20	1.17 ± 0.15	3.67 ± 0.22	1.49 ± 0.11	0.89 ± 0.17	0.92 ± 0.04	1.02 ± 0.10
*CLB6*	7.57 ± 1.28	18.06 ± 1.64	1.79 ± 0.26	1.54 ± 0.21	3.00 ± 0.37	1.63 ± 0.19	0.84 ± 0.13	1.06 ± 0.13	0.77 ± 0.05
*PGK1*	0.91 ± 0.08	0.66 ± 0.05	0.88 ± 0.08	1.36 ± 0.14	0.67 ± 0.08	0.91 ± 0.07	0.94 ± 0.03	0.70 ± 0.08	0.80 ± 0.08

**Table 2:** Comparative expression levels of *CLB* and *CLN* genes from the microarray data (GEO accession number GSE198743). The data show the relative microarray values of each gene obtained from wild-type and the *ccr4Δ* mutant strains. These values were normalized against their corresponding wild-type value which reflects the fold change in expression (values in parenthesis).

GENE	wild-type	*ccr4Δ*
*CLB1*	5902.6 (1)	15973 (2.71)
*CLB2*	4399.6 (1)	8455 (1.92)
*CLB3*	11321.7 (1)	12249.2 (1.08)
*CLB4*	3163 (1)	6157.5 (1.95)
*CLB5*	6176.6 (1)	7216.7 (1.17)
*CLB6*	2147.7 (1)	8633.2 (4.02)
*CLN1*	20929.4 (1)	19665.2 (0.94)
*CLN2*	5327.4 (1)	4789.2 (0.90)
*CLN3*	3867.8 (1)	3119.8 (0.81)
*ACT1*	150502.4 (1)	166707.8 (1.11)
*PGK1*	263333.2 (1)	260814 (0.99)

**Fig 3:**
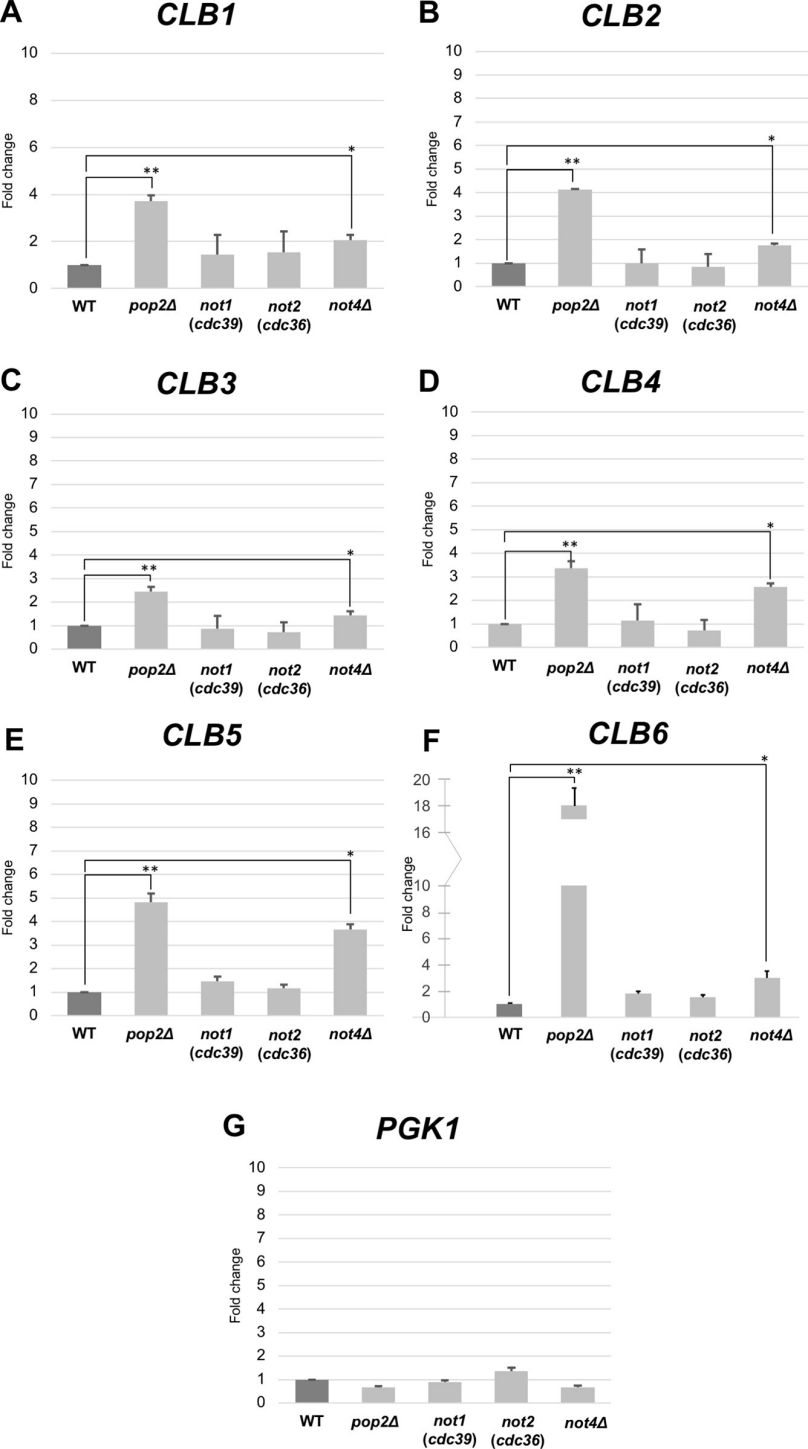
Expression of *CLB1*, *CLB2*, *CLB3*, *CLB4*, *CLB5*, and *CLB6* in wild-type, *pop2Δ*, *not1*, *not2*, and *not4Δ* mutant cells. The mRNA levels of *CLB1* (A), *CLB2* (B), *CLB3* (C), *CLB4* (D), *CLB5* (E), and *CLB6* (F) in *pop2*Δ, *not1*Δ, *not2*Δ, and *not4*Δ mutant strains growing in YPD media relative to the wild-type strain. *PGK1* (G) was used as a control. mRNA levels were quantified by qRT-PCR analysis, and the relative mRNA levels were calculated using 2^-ΔΔCt^ method normalized to *ACT1* reference gene. The data show mean ± SEM (n = 3) of fold change of mRNA level from wild-type cells at 4 h of culture in YPD. *P < 0.05, **P < 0.01 as determined by Tukey’s test.

Ccr4 is a catalytic subunit of the Ccr4-Not deadenylase complex [8]. To investigate whether other components of the Ccr4-Not complex are involved in the increase of the *CLB* mRNA levels, we examined endogenous mRNA levels of *CLB1-6* in the *pop2Δ*, *not1* (*cdc39*), *not2* (*cdc36*), and *not4Δ* mutant cells. Pop2, also known as Caf1, is an exonuclease of the DEDD super family that is another catalytic subunit of the Ccr4-Not deadenylase complex, Not1/Cdc39 is a scaffold protein, Not2/Cdc36 is one of core proteins, and Not4 is an E3 ubiquitin ligase [8]. As expected from the observation that the *pop2Δ* mutant is similar to the *ccr4*Δ mutant [34,35], all *CLB1-6* mRNA levels were increased in the *pop2*Δ mutant compared to those in wild-type cells (Fig 3). Similar to the *ccr4Δ* mutant, the increase of *CLB6* mRNA level, approximately 18-fold increase, was most prominent. The *CLB1*, *CLB2*, and *CLB5* mRNA levels were increased about 4 to 5-fold, and the *CLB3* and *CLB4* mRNA levels were increased about 3-fold. On the other hand, some *CLB* mRNA levels were slightly increased in the *not4Δ* mutant, and no *CLB* mRNA level was increased in the *not1*Δ or *not2*Δ mutant (Fig 3, Table 1). Thus, two catalytic subunits of the Ccr4-Not complex, Ccr4 and Pop2, are mainly involved in the levels of *CLB* mRNAs.

### The *CLB6* mRNA level was significantly increased in the *ccr4Δ* mutant in synchronized culture

**Fig 4:**
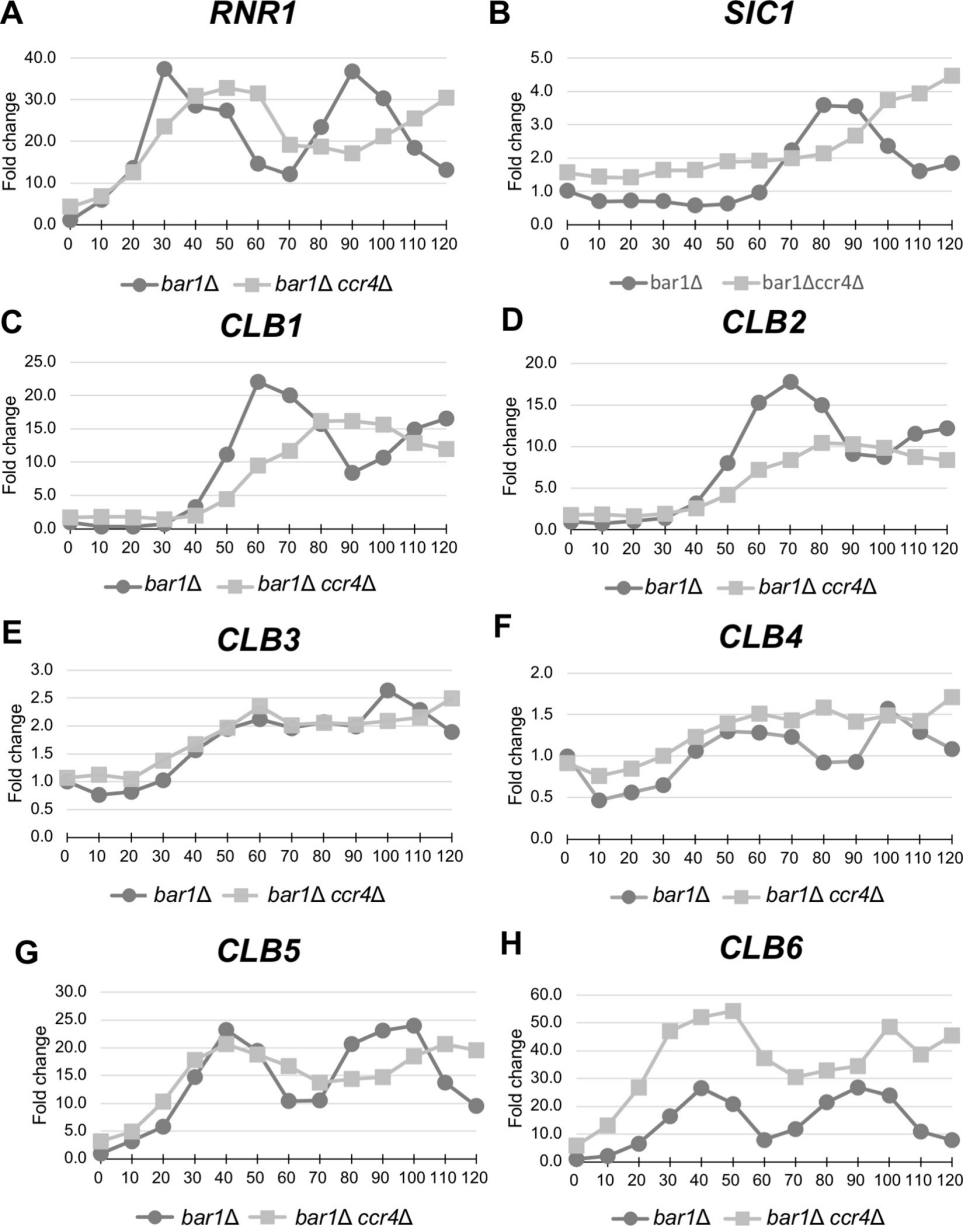
The cell-cycle regulated expression of *CLBs* in the *ccr4Δ* mutant. The qRT-PCR analysis data of RNR1 mRNA, SIC1 mRNA, and CLB mRNAs in the cell cycle synchronized bar1Δ mutant (black circle) and bar1Δ ccr4Δ mutant (gray square). Cell cycle was arrested in G1 phase by α-factor, and, after release, cells were collected from 0min (just before releasing) to 120min. The fold change of RNR1 mRNA, S phase marker, (A) and SIC1 mRNA, late M phase marker, (B) show cell cycle successfully progress both in the bar1Δ mutant and the bar1Δ puf5Δ mutant. The fold change of B-type cyclin mRNAs, CLB1 mRNA (C), CLB2 mRNA (D), CLB3 mRNA (E), CLB4 mRNA (F), CLB5 mRNA (G), CLB6 mRNA (H) are presented.

*CLBs* expression is known to be regulated during the cell cycle [1,14,15]. The increase in *CLBs* mRNA levels by the *ccr4Δ* mutation may be due to the *ccr4Δ* mutant being delayed in a particular phase during cell cycle. To examine this possibility, we analyzed a cell cycle progression of the *ccr4Δ* mutant and also examined the *CLB1-6* mRNA levels in synchronized cultures. For this purpose, we utilized the *MAT***a** *bar1Δ* and *MAT***a** *bar1Δ ccr4Δ* cells, in which α-factor protease Bar1 was absent [28]. The *MAT***a** *bar1Δ* and *MAT***a** *bar1Δ ccr4Δ* cells were arrested in G1 phase with α-factor. The cells were then released from the G1 arrest by several washes with fresh media and allowed to progress into the S phase. In the *bar1Δ* cell, the S phase marker *RNR1* peaked at 30 minutes, which was considered to be the S phase (Fig 4A). *CLB5* and *CLB6* also peaked at this time (Fig 4G and 4H). The peak of *RNR1* of the *bar1Δ ccr4Δ* mutant was slightly delayed compared to that of the *bar1Δ* strain and reached the peak at about 50 minutes (Fig 4A). The peak of the M phase marker, *SIC1*, in the *bar1Δ ccr4Δ* mutant was also delayed in comparison with that in the *bar1Δ* strain (Fig 4B).

In these synchronous cultures, the *CLB6* mRNA level, which had the largest increase in the *ccr4Δ* mutant in the asynchronous culture (Fig 2F), was significantly increased in the *bar1Δ ccr4Δ* mutant compared to those in the *bar1Δ* cells over the cell cycle (Fig 4H). The *CLB4* mRNA level was a little higher in the *bar1Δ ccr4Δ* mutant than those in the *bar1Δ* cells (Fig 4F). On the other hand, there were no significant increases in the *CLB1*, *CLB2*, *CLB3*, and *CLB5* mRNA levels in the synchronous *bar1Δ ccr4Δ* mutant (Fig 4C, 4D, 4E and 4G). Thus, the increase in *CLBs* mRNA levels by the *ccr4Δ* mutation in asynchronous culture seemed to be partly due to the *ccr4Δ* mutant being delayed in cell cycle. However, since the mRNA levels of *CLB6* and *CLB4* were elevated in the *bar1Δ ccr4Δ* mutant over the cell cycle, it was also likely that mRNA degradation by Ccr4 regulates *CLB* levels.

**Fig 5:**
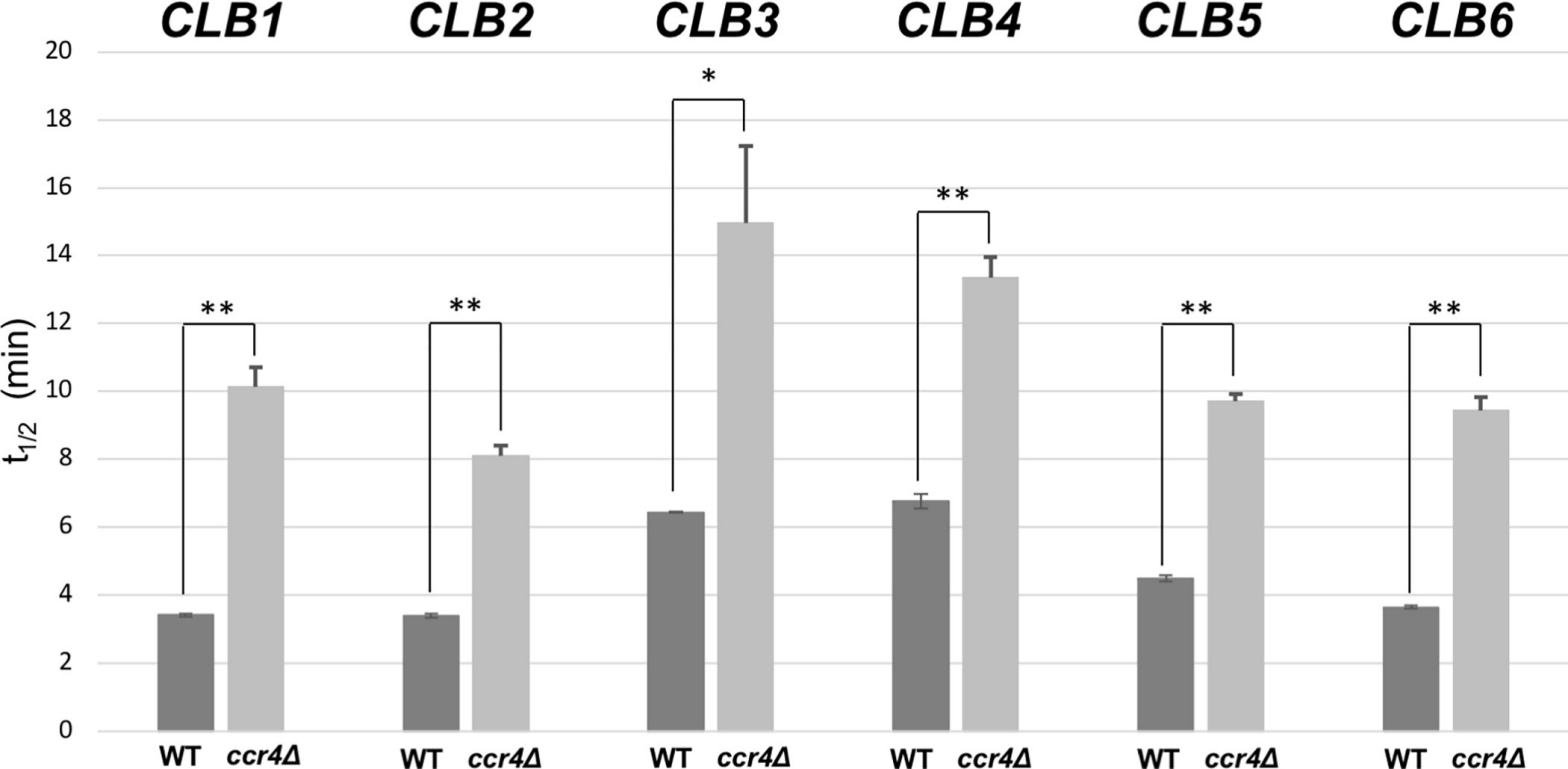
The half-lives of *CLB1-CLB6* mRNAs in wild-type and the *ccr4Δ* mutant. The *bar1*Δ and the *bar1*Δ *ccr4*Δ mutant cells were pre-cultured in YPD medium at 28°C overnight, and transferred into fresh YPD medium, and cultured until mid-exponential phase. Then, thiolutin was added, and samples were collected by centrifuge at 0, 1, 2, 5, 10, 20, 40, 80 minutes after exposure to thiolutin. The mRNA levels at each time point were determined by qRT-PCR. The half-lives (t_1/2_) were calculated using Microsoft Excel. *P < 0.05, **P < 0.01 as determined by Tukey’s test.

To examine whether Ccr4 is involved in mRNA degradation of *CLB* mRNAs, we analyzed the half-lives of *CLB* mRNAs after transcription inhibition with thiolutin (Fig 5). The half-lives of *CLB1-6* mRNAs in wild-type cells were 3 to 7 min, and those were clearly extended in the *ccr4Δ* mutant (Fig 5). It was surprising that the effects of the *ccr4Δ* mutation on the mRNA half-lives was similar in all *CLB1-6* mRNAs. The significant increase of the *CLB6* mRNA levels may be caused by the multiple effects of the *ccr4Δ* mutation, including mRNA degradation, cell cycle delay, or transcription.

**Fig 6:**
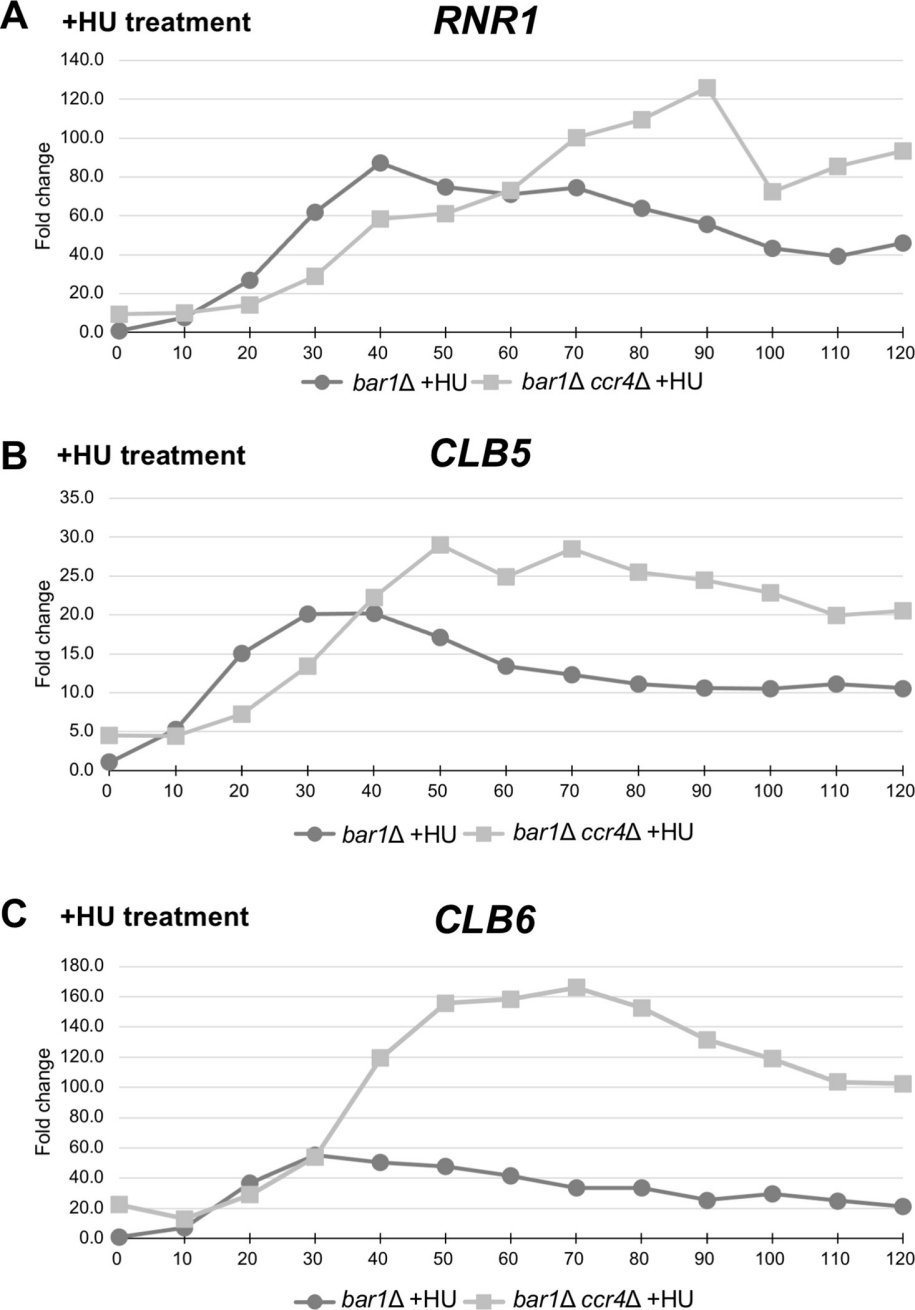
The cell-cycle regulated expression of *CLBs* in the *ccr4Δ* mutant. The qRT-PCR analysis data of RNR1, CLB5, and CLB6 mRNAs in the cell cycle synchronized bar1Δ mutant (black circle) and bar1Δ ccr4Δ mutant (gray square). Cell cycle was arrested in G1 phase by α-factor, and, after releasing in the presence of HU, cells were collected from 0 min (just before releasing) to 120 min. The fold change of RNR1 mRNA, S phase marker, (A) and SIC1 mRNA, late M phase marker, (B) show cell cycle successfully progress both in the bar1Δ mutant and the bar1Δ puf5Δ mutant. The fold change of B-type cyclin mRNAs, CLB1 mRNA (C), CLB2 mRNA (D), CLB3 mRNA (E), CLB4 mRNA (F), CLB5 mRNA (G), CLB6 mRNA (H) are presented.

*CLB6* expression is reported to be changed by various stresses, such as replication stress [30]. Then we examined whether the *ccr4Δ* mutation affects the *CLB6* mRNA levels in the replication stress condition. The MAT**a** *bar1Δ* and MAT**a** *bar1Δ ccr4Δ* cells synchronized in G1 with α-factor were synchronously released into the S phase in the presence of hydroxy urea (HU), a reagent that generates replication stress by depleting the pool of dNTPs. As shown in Fig 6C, the *CLB6* mRNA level peaked at 30 min in the *bar1Δ* cells and was kept at a high level. The *CLB6* mRNA levels were increased more in the *bar1Δ ccr4Δ* mutant (Fig 6C). While the *CLB5* mRNA level was not increased in the *bar1Δ ccr4Δ* mutant without replication stress (Fig 4G), the *CLB5* mRNA level was also increased in the HU-treated *bar1Δ ccr4Δ* mutant cells (Fig 6B). Thus, Ccr4 seems to contribute the *CLB5* and *CLB6* mRNA levels in replication stress condition.

### The Clb protein levels are not significantly increased in the *ccr4Δ* mutant

**Fig 7:**
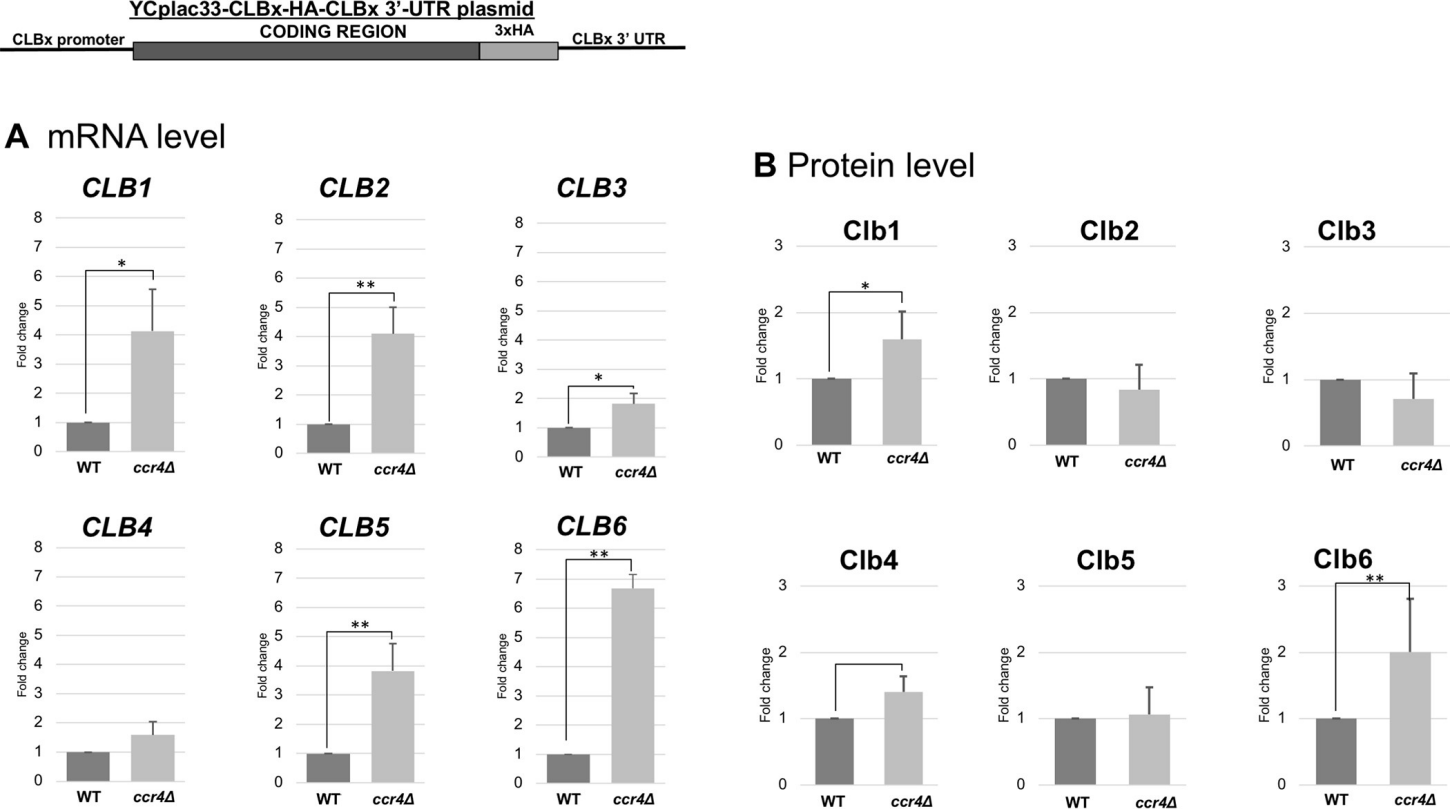
Expression of *CLB1-HA*, *CLB2-HA*, *CLB3-HA*, *CLB4-HA*, *CLB5-HA*, and *CLB6-HA* in wild-type and *ccr4Δ* mutant cells harboring the *CLBx-HA-CLBx 3’-UTR* plasmid. The mRNA (A) and protein (B) levels of *CLB1-HA*, *CLB2-HA*, *CLB3-HA*, *CLB4-HA*, *CLB5-HA*, and *CLB6-HA* in wild-type (WT) and *ccr4*Δ mutant cells growing in SC-ura media. The strains harboring the *CLBx-HA-CLBx 3’ UTR* plasmids were grown at 28°C. mRNA levels were quantified by qRT-PCR analysis, and the relative mRNA levels were calculated using 2^-ΔΔCt^ method normalized to *ACT1* reference gene. The data show mean ± SEM (n = 3) of fold change of mRNA level from wild-type cells at 4 H of culture in SC-ura. Protein levels were quantified by preparing cell extracts collected at log phase (4 H) for immunoblotting with anti-HA and anti-Pgk1 antibodies where Pgk1 was used as the loading control. The data show mean ± SEM (n = 3) of fold change of pr level from wild-type cells at 4 H of culture in SC-ura. *P < 0.05, **P < 0.01 as determined by Tukey’s test.

To examine whether the increased *CLB* mRNA levels by the *ccr4Δ* mutation confer the increased Clb protein levels, we next constructed the *CLBx-HA-CLBx 3’ UTR* plasmids (Fig 7). Using these plasmids, we measured the *CLBx-HA* mRNA levels and the Clbx-HA protein levels in wild-type and the *ccr4Δ* mutant cells. We used a specific primer to detect *CLBx-HA* mRNA, but not endogenous *CLBx* mRNA. Generally, all the *CLB1-6-HA* mRNA levels were increased in the *ccr4Δ* mutant compared to those in wild-type cells (Fig 7A, *CLBx*-HA mRNA, Table 3). The ratios were somewhat different from the results of endogenous *CLB* mRNAs. The *CLB6-HA* mRNA level was increased about 7-fold; the *CLB1-HA*, *CLB2-HA*, and *CLB5-HA* mRNA levels were increased about 4-fold; the *CLB3-HA* and *CLB4-HA* mRNA levels were increased less than 2-fold. In contrast to the results of the increased mRNA levels, we found that the Clb1-6-HA protein levels were not significantly increased in the *ccr4Δ* mutant compared to those in wild-type cells (Fig 7B, Clb1-6-HA protein, Table 3). The Clb6-HA protein level was increased about 2-fold; the Clb1-HA and Clb4-HA protein levels were increased about 1.5-fold; the Clb2-HA, Clb3-HA. and Clb5-HA protein levels were not increased. These results indicated that Ccr4 contributes the levels of *CLB* mRNAs, but the Clb protein levels are not increased in the *ccr4Δ* mutant (summarized in Table 3).

**Table 3:** Summary of *ccr4*Δ*/*wild-type ratio of mRNA and protein levels on different gene constructs. All values written are standardized to their respective ccr4Δ/wild-type ratio.

Gene	CLBx-HA-CLBx 3’UTR		CLBx-HA-ADH1 3’UTR		MCM2-GFP CLBx 3’UTR -	
	mRNA	protein	mRNA	protein	mRNA	protein
*CLB1*	4.13 ± 1.76	1.60 ± 0.41	4.70 ± 0.90	1.24 ± 0.42	3.41 ± 0.87	14.88 ± 2.75
*CLB2*	4.09 ± 1.12	0.84 ± 0.37	2.67 ± 0.36	0.97 ± 0.14	4.32 ± 0.76	12.00 ± 2.63
*CLB3*	1.83 ± 0.41	0.71 ± 0.38	1.66 ± 0.46	0.89 ± 0.28	2.48 ± 0.80	5.36 ± 2.21
*CLB4*	1.59 ± 0.57	1.41 ± 0.23	2.74 ± 1.01	1.62 ± 0.56	2.60 ± 0.93	10.72 ± 4.85
*CLB5*	3.83 ± 1.16	1.06 ± 0.42	1.79 ± 0.59	0.96 ± 0.24	3.39 ± 0.46	8.43 ± 2.10
*CLB6*	6.67 ± 0.59	2.01 ± 0.80	3.29 ± 1.01	3.15 ± 1.31	6.48 ± 2.11	15.42 ± 3.30

### The coding sequences of *CLB* mRNAs are involved in the Ccr4-dependent regulation

**Fig 8:**
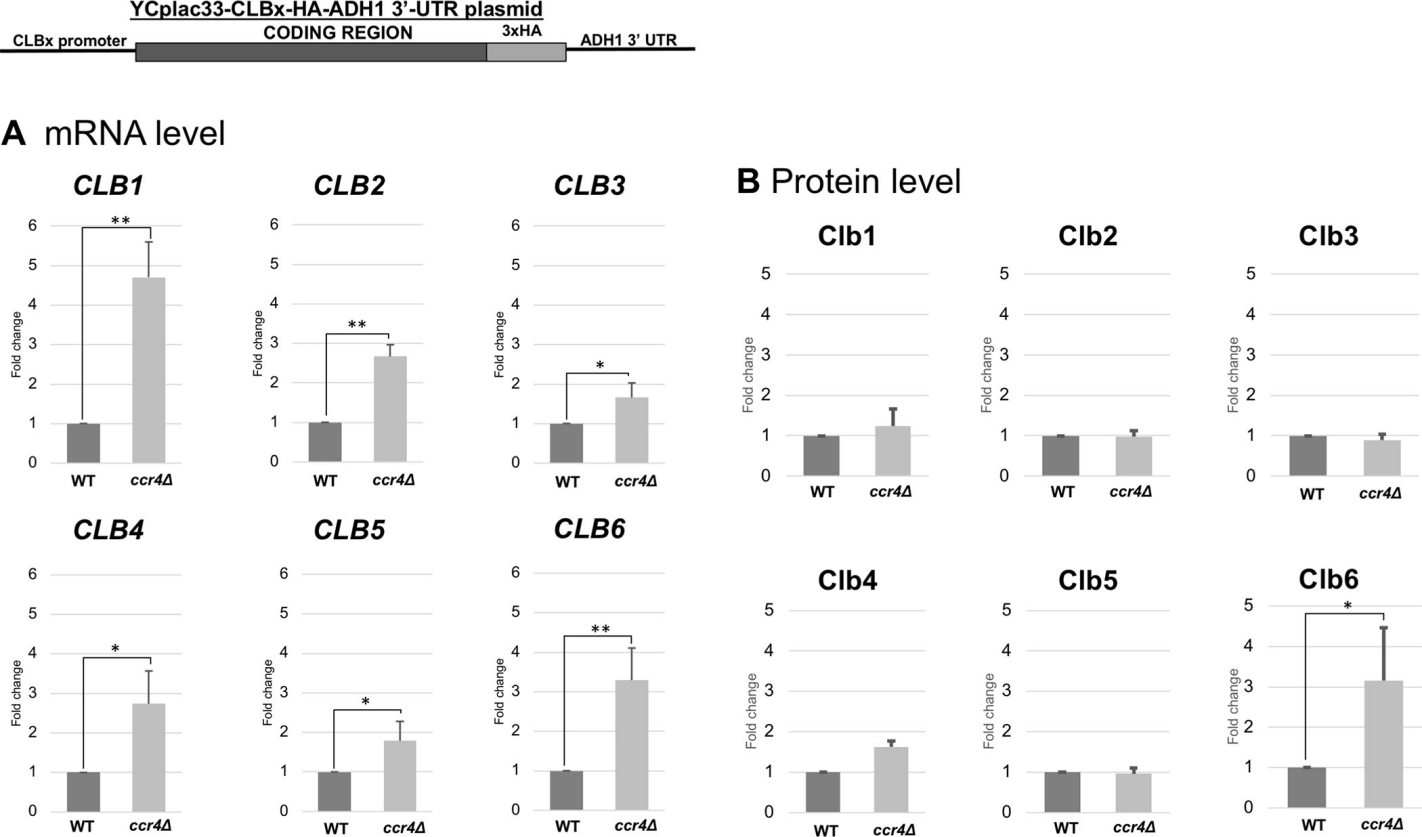
Expression of *CLB1-HA*, *CLB2-HA*, *CLB3-HA*, *CLB4-HA*, *CLB5-HA*, and *CLB6-HA* in wild-type and *ccr4Δ* mutant cells harboring the *CLBx-HA-ADH1 3’ UTR* plasmid. The mRNA (A) and protein (B) levels of *CLB1-HA*, *CLB2-HA*, *CLB3-HA*, *CLB4-HA*, *CLB5-HA* and *CLB6-HA* in wild-type (WT) and *ccr4Δ* mutant strain growing in SC-ura media. The strains harboring the *CLBx-HA-ADH1 3’ UTR* plasmid were grown at 28°C. mRNA levels were quantified by qRT-PCR analysis, and the relative mRNA levels were calculated using 2^-ΔΔCt^ method normalized to *ACT1* reference gene. The data show mean ± SEM (n = 3) of fold change of mRNA level from wild-type cells at 4 h of culture in Sc-ura. Protein levels were quantified by preparing cell extracts collected at log phase (4 H) for immunoblotting with anti-HA and anti-Pgk1 antibodies where Pgk1 was used as the loading control. The data show mean ± SEM (n = 3) of fold change of protein level from wild-type cells at 4 H of culture in SC-ura. *P < 0.05, **P < 0.01 as determined by Tukey’s test.

Although it was reported that mRNA decay is usually mediated at the 5’ UTR and 3’ UTR ends [36], we examined whether the coding sequences of *CLB* genes are involved in mRNA levels. This was done by constructing the *CLBx-HA-ADH1 3’ UTR* plasmids (Fig 8). The same method of estimating the mRNA level by qRT-PCR using the HA primers and protein level by HA tagging were done.

First, we examined the *CLBx-HA-ADH1 3’ UTR* mRNA levels in wild-type and the *ccr4Δ* mutant cells (Fig 8). All the *CLB1-6-HA-ADH1 3’ UTR* mRNA levels were increased in the *ccr4Δ* mutant compared to those in wild-type cells (Fig 8A, *CLBx*-HA mRNA). The *CLB1-HA-ADH1 3’ UTR* mRNA level was increased about 5-fold; the *CLB2-HA-ADH1 3’ UTR*, *CLB4-HA-ADH1 3’ UTR*, and *CLB6-HA-ADH1 3’ UTR* mRNA levels were increased about 3-fold; the *CLB3-HA-ADH1 3’ UTR* and *CLB5-HA-ADH1 3’ UTR* mRNA levels were increased with less than 2-fold.

When we compared the results of the *CLBx-HA-CLBx 3’ UTR* plasmids and the *CLBx-HA-ADH1 3’ UTR* plasmids (Figs 7A and 8A, Table 3), the increased fold of *CLB5* and *CLB6* mRNAs in *ccr4Δ* mutant was different. In the *CLBx-HA-CLBx 3’ UTR* plasmids, the *CLB6-HA* mRNA level was increased about 7-fold; *CLB5-HA* mRNA levels was increased about 4-fold (Fig 7A, Table 3). However, in the *CLBx-HA-ADH1 3’ UTR* plasmids, *CLB6-HA-ADH1 3’ UTR* mRNA levels was increased about 3-fold; *CLB5-HA-ADH1 3’ UTR* mRNA level was increased with less than 2-fold (Fig 8A, Table 3). These results suggest that the coding sequence of *CLB6*, but not that of *CLB5*, may have a role in mRNA level and that the *3’ UTRs* of *CLB5* and *CLB6* may have a role in mRNA level.

Then we examined the Clbx-HA protein levels from the *CLBx-HA-ADH1 3’ UTR* plasmids in wild-type and the *ccr4Δ* mutant cells (Fig 8B, Clb1-6-HA protein). The Clb6-HA protein level was increased about 3-fold; the Clb4-HA protein level was increased about 1.7-fold; the Clb1-HA, Clb2-HA, Clb3-HA and Clb5-HA protein levels were not increased.

These results indicated that the coding sequences of some *CLB* mRNAs are involved in the Ccr4-dependent regulation, but again the Clb protein levels are not simply dependent on the mRNA levels (summarized in Table 3).

### The 3’ UTR sequences of *CLB* mRNAs are involved in the Ccr4-dependent mRNA destabilization

**Fig 9:**
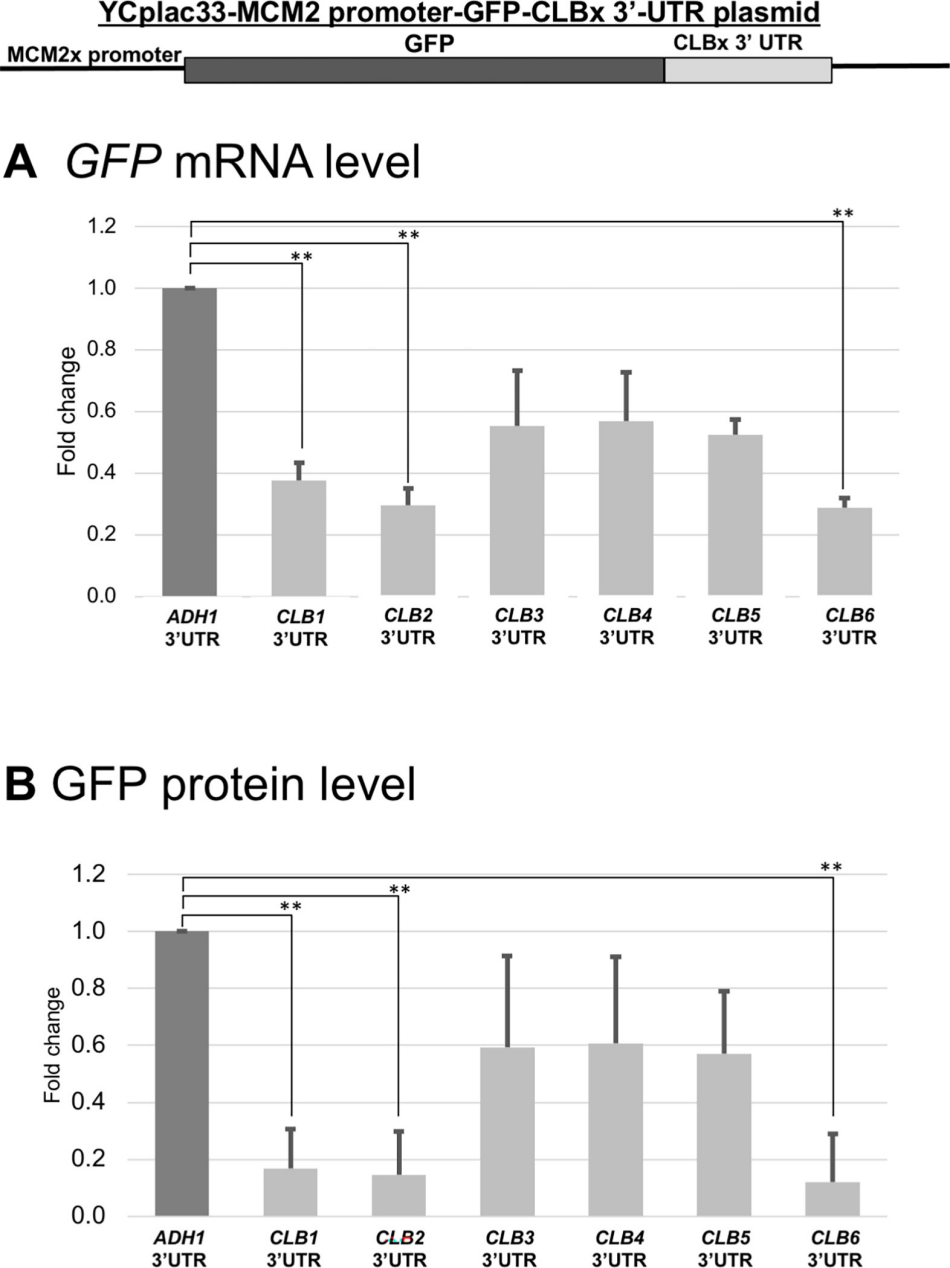
Expression of GFP mRNA and protein in wild-type strain harboring the *MCM2 promoter-GFP-CLBx 3’-UTR* plasmids. The mRNA (A) and protein (B) levels of in wild-type (WT) strains harboring the *MCM2 promoter-GFP-CLBx 3’ UTR* plasmids were grown at 30°C in SC-ura media. mRNA levels were quantified by qRT-PCR analysis, and the relative mRNA levels were calculated using 2^-ΔΔCt^ method normalized to *ACT1* reference gene. The data show mean ± SEM (n = 3) of fold change of mRNA level from wild-type cells at 4 H of culture in Sc-ura. Protein levels were quantified by preparing cell extracts collected at log phase (4 H) for immunoblotting with anti-GFP and anti-Pgk1 antibodies where Pgk1 was used as the loading control. The data show mean ± SEM (n = 3) of fold change of protein level from wild-type cells at 4 H of culture in SC-ura. *P < 0.05, **P < 0.01 as determined by Tukey’s test.

The 3’ UTR of an mRNA is reported to be crucial for degradation, and this is conserved throughout eukaryotes [36]. Here, we assumed that the 3’ UTRs of *CLB1-6* are also a key for its stability. To confirm this assumption, we constructed the *MCM2 promoter-GFP-CLBx 3’ UTR* plasmids which express the *GFP-CLBx 3’ UTR* mRNAs and GFP proteins from *MCM2* promoter (Fig 9). Mcm2 protein is present through the cell cycle [37], the *MCM2* promoter activity is not strong [38], and the changes in mRNA and protein levels are easy to detect. The different *CLBx* 3’ UTRs were compared with *ADH1* 3’ UTR by using GFP primers for qRT-PCR and GFP antibody for Western blot.

First, we compared the *GFP-CLB1-6 3’ UTR* mRNA levels with the *GFP-ADH1 3’ UTR* mRNA level in wild-type cells. Generally, all the *GFP-CLB1-6 3’ UTR* mRNA levels were decreased than the *GFP-ADH1 3’ UTR* mRNA level, but its ratio differed in the genes (Fig 9A, *GFP* mRNA, Table 4). The *CLB1*, *CLB2*, and *CLB6 3’ UTRs* gave about 0.30-fold decrease; the *CLB3*, *CLB4*, and *CLB5 3’ UTRs* gave about 0.60-fold decrease. Then we examined the GFP protein levels from the *MCM2 promoter-GFP-CLBx 3’ UTR* plasmids in wild-type (Fig 9B, GFP protein, Table 4). In this case, it is consistent with the results of mRNA levels, the GFP protein levels from the *CLB1-6 3’ UTR* constructs were decreased than that of the *ADH1 3’UTR* construct. The *CLB1*, *CLB2*, and *CLB6 3’ UTRs* gave about 0.20-fold decrease of GFP protein level; the *CLB3*, *CLB4*, and *CLB5 3’ UTRs* gave about 0.60-fold decrease of GFP protein level. These decreases of GFP protein levels were correlated with GFP mRNA levels (Fig 9A and 9B, Table 4). These results indicated that the *CLBx 3’UTRs* have destabilizing sequence and the instability caused by the 3’ UTR sequence simply contributes the decrease of protein level in the GFP reporter construct.

**Table 4:** Summary of GFP mRNA and protein levels of different 3’-UTRs of *CLB*. All gene constructs were transformed into wild-type strain and standardized on *ADH1* 3’-UTR.

	mRNA	protein
*CLB1*	0.377 ± 0.06	0.168 ± 0.139
*CLB2*	0.295 ± 0.056	0.146 ± 0.151
*CLB3*	0.553 ± 0.179	0.594 ± 0.320
*CLB4*	0.570 ± 0.158	0.608 ± 0.303
*CLB5*	0.525 ± 0.050	0.571 ± 0.220
*CLB6*	0.288 ± 0.032	0.120 ± 0.171

**Fig 10:**
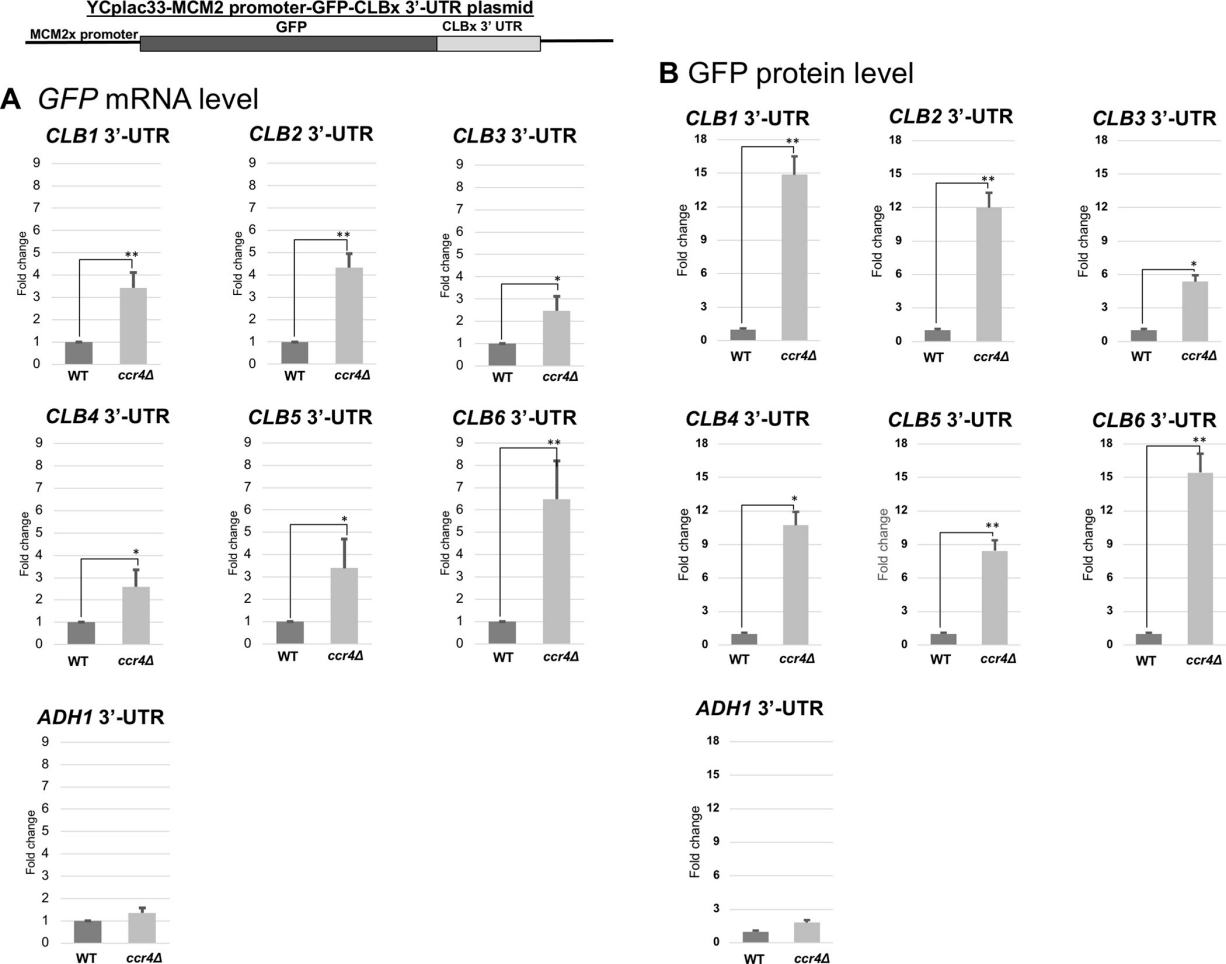
Expression of GFP mRNA and protein in wild-type and *ccr4Δ* mutant cells harboring the *MCM2 promoter-GFP-CLBx 3’ UTR* plasmids. The mRNA (A) and protein (B) levels of GFP in wild-type (WT) and *ccr4*Δ strains harboring the *MCM2 promoter-GFP-CLBx 3’ UTR* plasmids were grown at 28°C in SC-ura media. mRNA levels were quantified by qRT-PCR analysis, and the relative mRNA levels were calculated using 2^-ΔΔCt^ method normalized to *ACT1* reference gene. The data show mean ± SEM (n = 3) of fold change of mRNA level from wild-type cells at 4 H of culture in Sc-ura. Protein levels were quantified by preparing cell extracts collected at log phase (4 H) for immunoblotting with anti-GFP and anti-Pgk1 antibodies where Pgk1 was used as the loading control. The data show mean ± SEM (n = 3) of fold change of protein level from wild-type cells at 4 H of culture in SC-ura. *P < 0.05, **P < 0.01 as determined by Tukey’s test.

To investigate mRNA stability control of *CLB1-6 3’UTR* sequence involves Ccr4, we examined the *GFP-CLBx 3’ UTR* mRNA levels and the GFP protein levels in wild-type and the *ccr4Δ* mutant cells (Fig 10, Table 3). All *GFP-CLBx 3’ UTR* mRNA levels were increased in the *ccr4Δ* mutant compared to those in wild-type cells, while the *GFP-ADH1 3’ UTR* mRNA level was not (Fig 10A, GFP mRNA, Table 3). The *GFP-CLB6 3’ UTR* was about 6.5-fold increase in the *ccr4Δ* mutant compared to that in wild-type cells; the *GFP-CLB2 3’ UTR* was about 4-fold increased; the *GFP-CLB1 3’ UTR* and *GFP-CLB5 3’ UTR* were about 3-fold increased; the *GFP-CLB3 3’ UTR* and *GFP-CLB4 3’ UTR* were about 2.5-fold increased. Consistent with the results of mRNA levels, the GFP protein levels from the *MCM2 promoter-GFP-CLBx 3’ UTR* constructs were increased in the *ccr4Δ* mutant than those in wild-type cells (Fig 10B, GFP protein). The GFP protein level from the *GFP-ADH1 3’ UTR* construct was similar in the *ccr4Δ* mutant and wild-type cells (Fig 10B, GFP protein, Table 3). The GFP protein levels derived from *GFP-CLB1 3’ UTR* and *GFP-CLB6 3’ UTR* were about 15-fold-increased in the *ccr4Δ* mutant compared to that in wild-type cells; the GFP protein levels derived from *GFP-CLB2 3’ UTR* and *GFP-CLB4 3’ UTR* were about 12-fold-increased; the GFP protein level derived from *GFP-CLB5 3’ UTR* was about 9-fold-increased; the GFP protein level derived from *GFP-CLB3 3’ UTR* was about 6-fold-increased (Fig 10B, GFP protein, Table 3). These results indicated that mRNA instability mediated by *CLB1-6 3’ UTR* involves Ccr4 and that the instability caused by the 3’ UTR sequence simply contributes the decrease of protein level in the GFP reporter construct (summarized in Table 3). This result is different from that of the *CLBx-HA* plasmid (summarized in Table 3).

### The *CLB6* coding sequence contains the three regions for mRNA destabilization

**Fig 11:**
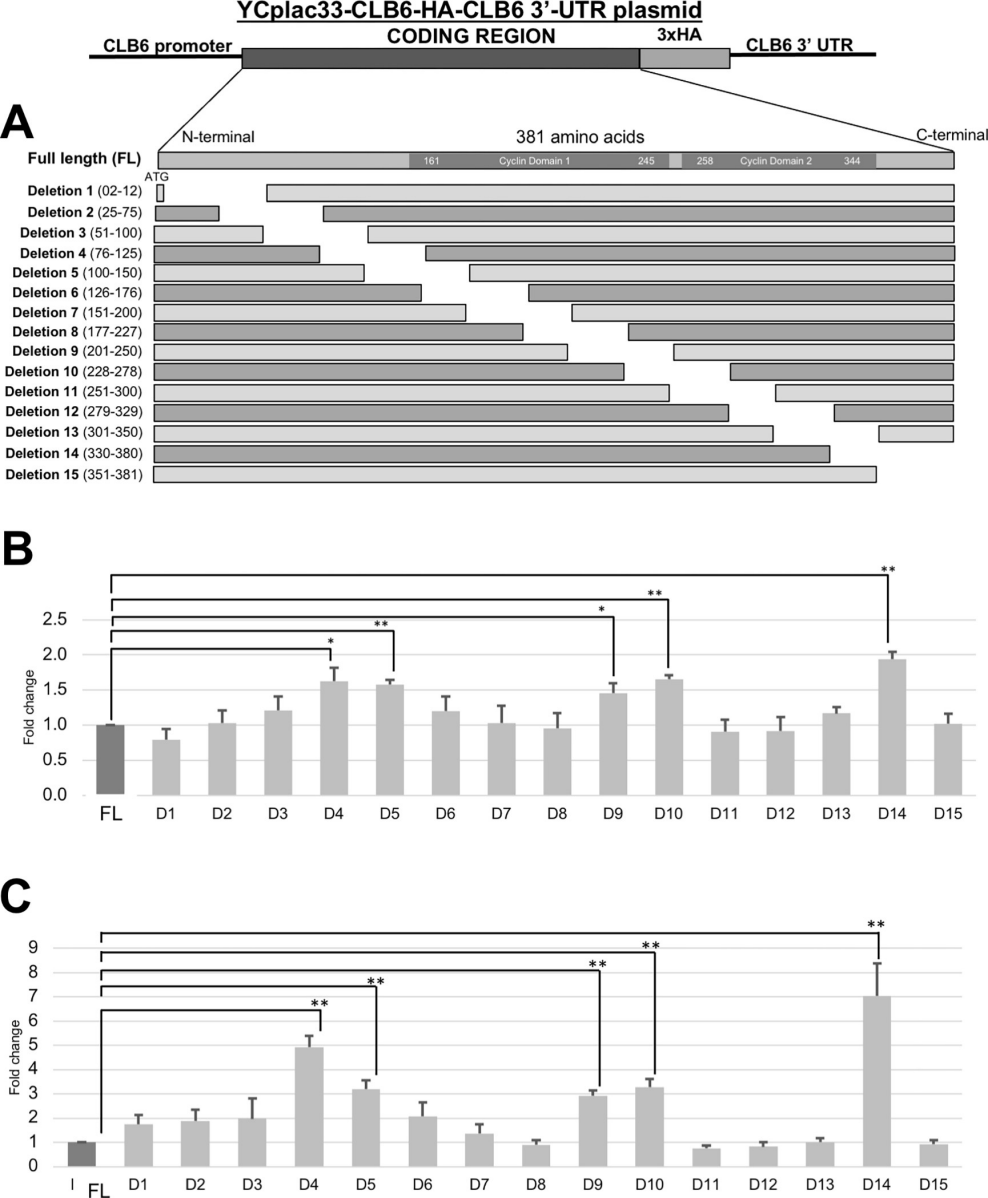
Deletion analysis of the *CLB6* coding sequence. Deletion sequences were done by deletion 10 amino acid base pairs per region (Deletion 1 to 15) relative to the ND (no deletion). The Cyclin Domain 1 and 2 are highlighted as grey (A). The mRNA (B) and protein (C) levels comparing the full-length and deletion strains transformed in wild-type strain grown in SC-ura media at 30°C. Protein levels are prepared on two gels from D1-7 and 8–15 and were quantified relative to the ND gene. mRNA levels were quantified by qRT-PCR analysis, and the relative mRNA levels were calculated using 2^-ΔΔCt^ method normalized to *ACT1* reference gene. The data show mean ± SEM (n = 3) of fold change of mRNA level from wild-type cells at 4 H of culture in SC-ura. Protein levels were quantified by preparing cell extracts collected at log phase (4 H) for immunoblotting with anti-HA and anti-Pgk1 antibodies where Pgk1 was used as the loading control. The data show mean ± SEM (n = 3) of fold change of protein level from wild-type cells at 4 H of culture in SC-ura. *P < 0.05, **P < 0.01 as determined by Tukey’s test.

From the above results of the *CLB6-HA-ADH1 3’ UTR* plasmid, we have shown that *CLB6* mRNA and protein levels were most increased in the *ccr4*Δ mutant (Fig 8A and 8B, Table 3). We suspected that a certain sequence within the coding region is responsible for the destabilization of the *CLB6* mRNA. To determine the sequence for mRNA instability, we constructed a series of deletions in the *CLB6-HA-CLB6 3’ UTR* gene construct, deletions of 150 base pairs corresponding 50 amino acids starting from the 2^nd^ bp after the start codon with a total of 15 deletions (Fig 11A). We then compared the mRNA level by qRT-PCR and protein level by western blot using the HA tag.

First, we investigated the mRNA level. Our results showed that certain deletion sequences significantly increased the *CLB6* mRNA by 1.5 to 2-fold. These were the sequences, D4 and D5, D9 and D10, and D14 (Fig 11B). As for the other deletions, we suspect that they are not crucial for destabilization as they showed incomparable results with that of the complete sequence.

Next, we investigated the protein level. Unsurprisingly, the same deletion sequences with the increase in mRNA level also showed an increase in protein level. That is, D4 and D5, D9 and D10, and D14 by 3 to 9-fold increase (Fig 11C). The other deletions did not show any significant difference with the complete sequence. From these data, we were able to deduce the certain sequences within the coding region have an immense effect on the destabilization of *CLB6*.

### The 3’ UTR sequence is crucial for *CLB6* expression

**Fig 12:**
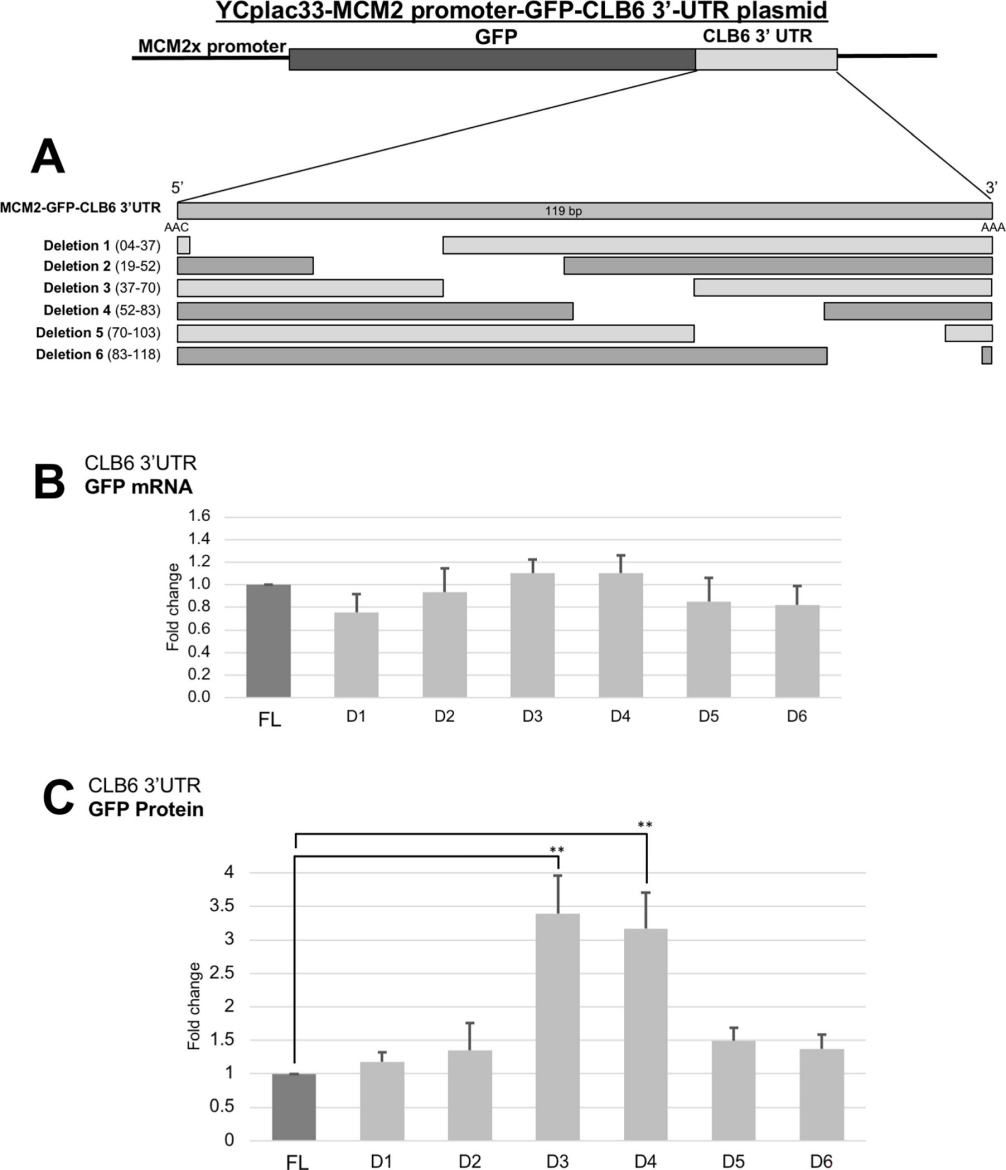
Deletion analysis of the *CLB6* 3’ UTR. Deletion sequences were done by ~30 base pairs deletion per region (Deletion 1 to 6) starting from the 5’ end after the GFP relative to the full-length. The mRNA (B) and protein level (C) in wild-type strain harboring gene construct of *MCM2-GFP-CLB6 3’ UTR* were grown at 30°C in SC-ura media. mRNA levels were quantified by qRT-PCR analysis and were calculated using 2^-ΔΔCt^ method normalized to *ACT1* reference gene. Protein levels were quantified by preparing cell extracts collected at log phase (4 H) for immunoblotting with anti-GFP and anti-Pgk1 antibodies where Pgk1 was used as the loading control. It was plotted as the fold change relative to the ‘no deletion strain’ cells at 4H of culture. The data show mean ± SEM (n = 3) *P < 0.05, **P < 0.01 as determined by Tukey’s test.

Comparing the *CLB1-6* 3’ UTR, we have shown that *CLB6* 3’ UTR was significantly decreased by Ccr4 both on the mRNA and protein level (Fig 10A and 10B, Table 4). This implicated that *CLB6* 3’ UTR contains a sequence responsible for proper degradation. To test this hypothesis, we constructed multiple deletions at the 3’ UTR of *CLB6*. We used the *MCM2* promoter-*GFP-CLB6 3’ UTR* plasmid and prepared deletions of 30 base’s starting from the 2^nd^ bp after the start of 3’ UTR (Fig 12A). Then we measured both the mRNA level and protein level by qRT-PCR and western blot, respectively. Comparing the mRNA level among the deletion constructs, there was no significant difference in all deletion constructs (Fig 12B). Though, D3 and D4 showed slightly higher mRNAs which were almost close to being significant. The lowest was deletion 1 which was close to the undeleted sequence. However, the increase was shown at the protein level on D3 and D4 (Fig 12C). This may further explain that the control mediated by the 3’ UTR only happens at the protein level and not on the mRNA. This elucidates that those regions have a specific sequence critical for the degradation of *CLB6*.

### The increase of *CLB6* expression in the *ccr4Δ* mutant is not dependent on the Puf protein

**Fig 13:**
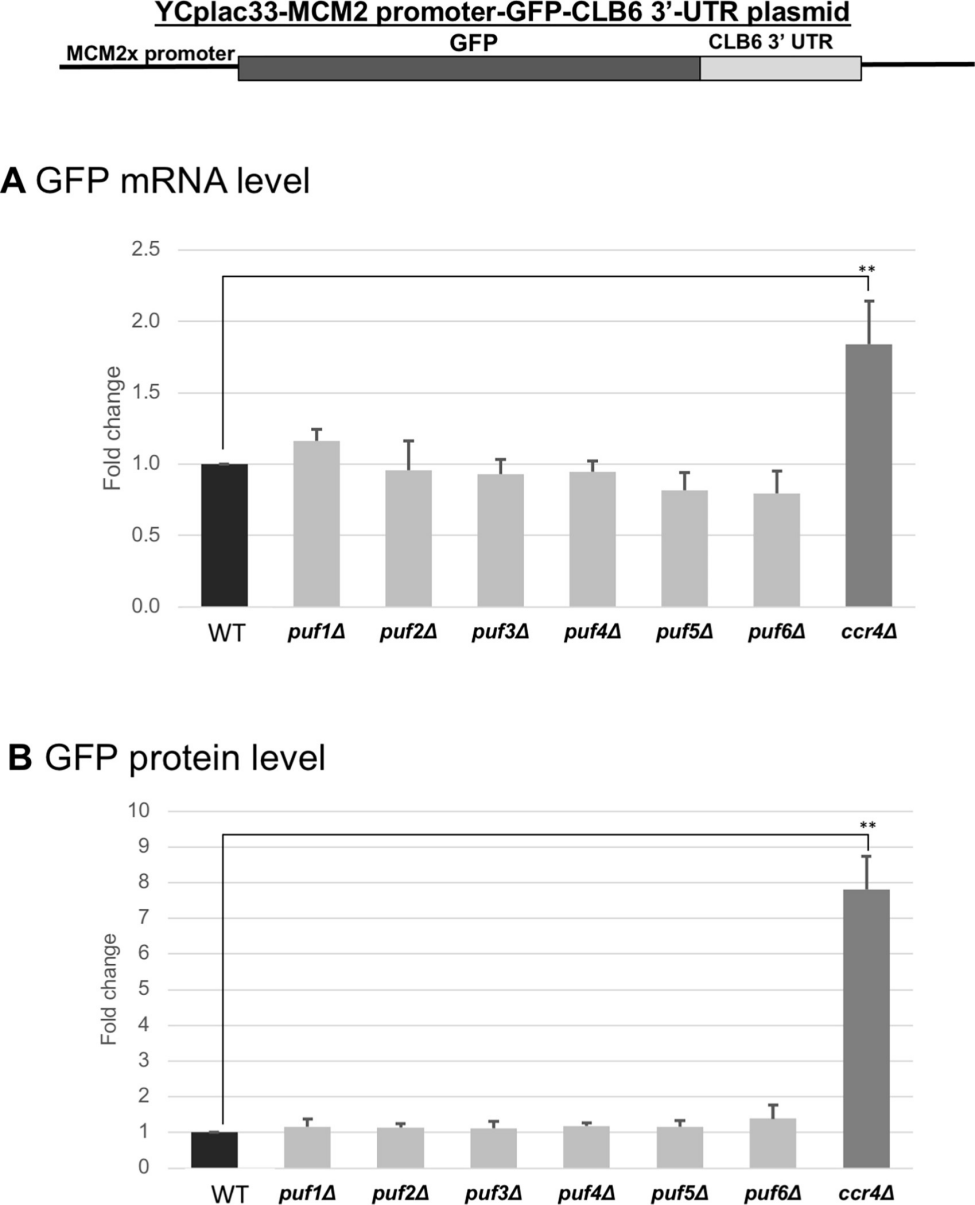
Expression of GFP on gene construct of MCM2-GFP-CLB6 3’-UTR with *pufΔ* mutants. The mRNA (A) and protein (B) levels of GFP in wild-type (WT), *puf1*Δ, *puf2*Δ, *puf3*Δ, *puf4*Δ, *puf5*Δ, *puf6*Δ, and *ccr4*Δ strains harboring the gene constructs of *MCM2-GFP-CLB6 3’UTR* grown at 28°C in SC-ura. mRNA levels were quantified by qRT-PCR analysis, and the relative mRNA levels were calculated using 2^-ΔΔCt^ method normalized to *ACT1* reference gene. The data show mean ± SEM (n = 3) of fold change of mRNA level from wild-type cells at 4 H of culture in SC-ura. Protein levels were quantified by preparing cell extracts collected at log phase (4 H) for immunoblotting with anti-GFP and anti-Pgk1 antibodies where Pgk1 was used as the loading control. The data show mean ± SEM (n = 3) of fold change of protein level from wild-type cells at 4 H of culture in SC-ura. *P < 0.05, **P < 0.01 as determined by Tukey’s test.

Puf family RNA-binding proteins are known to recruit the Ccr4-Not deadenylase complex to the mRNAs promote decay [9,39,40]. From the previous results, we have shown that *CLB6* was significantly destabilized by Ccr4 on both the coding and 3’ UTR regions. Using the *MCM2* promoter-*GFP-CLB6 3’ UTR* plasmid, we investigated the *GFP* mRNA and protein level on the deletion strains of *PUF1-6* genes and compare them with the *ccr4*Δ mutant. Results showed that there was no significant increase for both the mRNA (Fig 13A) and protein level (Fig 13B) on the *puf1-6*Δ mutants. These results suggest that Puf proteins are not involved in the Ccr4-dependent mRNA destabilization of *CLB6*.

### The deletion of *WHI3* showed an increased level of endogenous *CLB* mRNA

**Fig 14:**
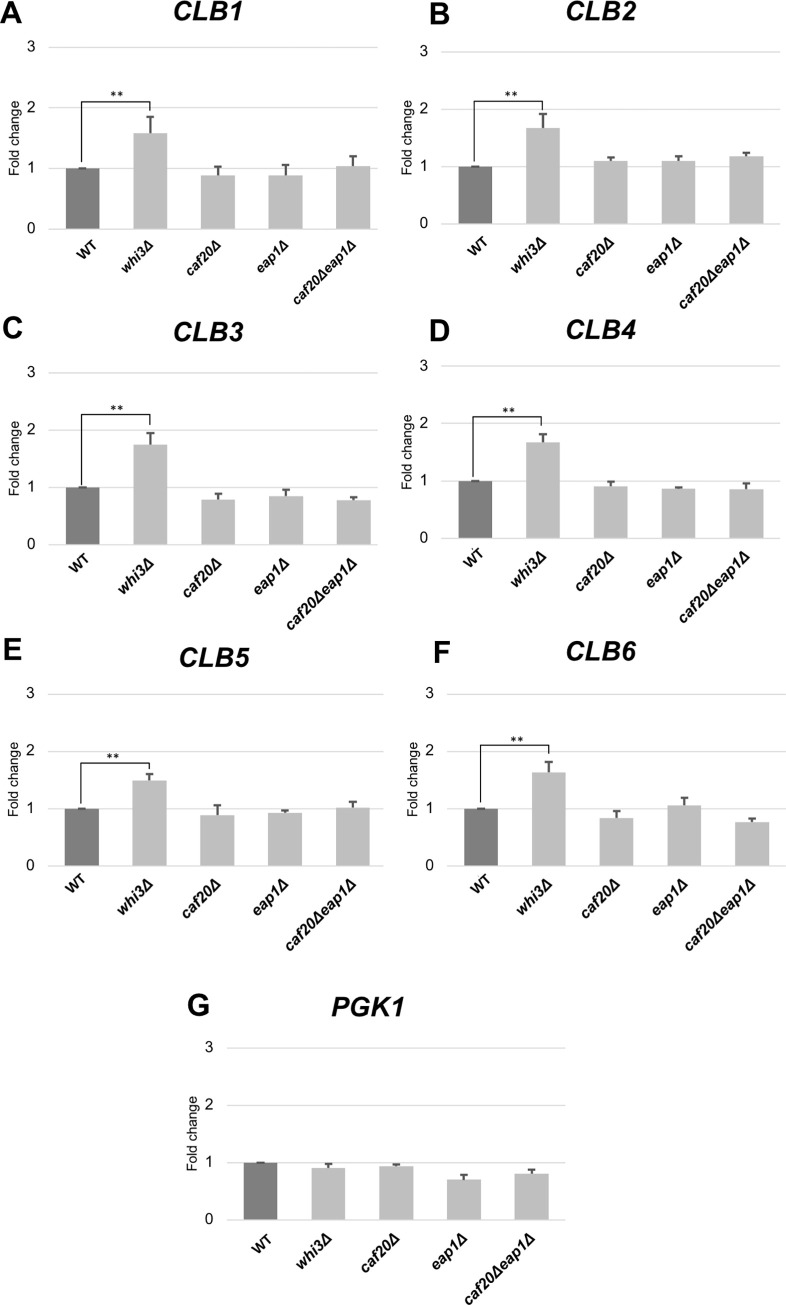
Expression of *CLB1*, *CLB2*, *CLB3*, *CLB4*, *CLB5*, and *CLB6* in *whi3Δ*, *caf20Δ*, *eap1Δ* and *caf20Δ eap1Δ* strains. The mRNA levels of *CLB1* (A), *CLB2 (B)*, *CLB3 (C)*, *CLB4 (D)*, *CLB5 (E)*, and *CLB6 (F)* in *ccr4*Δ mutant strain growing in YPD medium relative to the wild-type strain. *PGK1 (G)* was used as positive control. mRNA levels were quantified by qRT-PCR analysis, and the relative mRNA levels were calculated using 2^-ΔΔCt^ method normalized to *ACT1* reference gene. The data show mean ± SEM (n = 3) of fold change of mRNA level from wild-type cells at 4 H of culture in YPD. *P < 0.05, **P < 0.01 as determined by Tukey’s test.

To further investigate the possible involvement of other RNA-binding proteins on the expression of *CLB* genes, we prepared several deletion strains, *whi3Δ*, *caf20Δ*, and *eap1Δ* mutants. Whi3 and Caf20 are reported to interact with *CLB* mRNA [41,42]. Eap1 was also selected because Eap1 is an eIF4E-binding protein, similar to Caf20 [43]. Using the same primers for the endogenous mRNA of *CLB1* to *CLB6*, we measured the mRNA levels. Comparing the different deletion strains, we found that the *whi3*Δ mutant showed a significant increase in all *CLB* mRNAs (Fig 14, Table 1). These results suggest that Whi3 may have a role in mRNA destabilization of *CLB* mRNAs.

## Discussion

### Ccr4 is involved in the expression of *CLB* genes

In this study, we examined *CLB1-6* mRNA levels in the *ccr4Δ* mutant. The first experiment was to investigate the significance of Ccr4 on the endogenous *CLB1-6* mRNAs. The data in Fig 1 showed that all *CLB1-6* mRNA levels were increased in the *ccr4Δ* mutant. Although the increase in mRNA happens for all the *CLB1-6* genes, it was most prominent in *CLB6*. While the levels of *CLB1-6* mRNAs are increased in the *ccr4Δ* mutant compared to wild-type, the mRNA levels of G1 cyclin genes, *CLN1-3*, are not increased in the *ccr4Δ* mutant (Table 1). Recent analyses [44] showed that poly A tail lengths of *CLB1-6* mRNA are increased in the *ccr4Δ* mutant (poly A tail lengths in wild-type/*ccr4Δ*, *CLB1*: 36.5/48.4, *CLB2*: 38.8/ 46.8, *CLB3*: 50.7/ 49.7, *CLB4*: 33.0/ 49.7, *CLB5*: 38.1/ 54.4, *CLB6*: 0.01/ 51.7). These data are consistent with our observation that *CLB1-6* mRNA levels are increased in the *ccr4Δ* mutants and that *CLB6* mRNA level is most affected by the *ccr4Δ* mutation. The *CLB6* mRNA level was also significantly increased in the *ccr4*Δ mutant in synchronized culture (Fig 3). On the other hand, the half-lives of *CLB1-CLB6* mRNAs are similar: 3 to 7 mins in wild-type, 10–15 mins in the *ccr4Δ* mutant. The significant increase of the *CLB6* mRNA level may be caused by the multiple effects of the *ccr4Δ* mutation, including mRNA degradation, cell cycle delay, or transcription.

We also examined whether the Clb1-6 protein levels were also affected by the *ccr4Δ* mutation. Unexpectedly, although the *CLB1-6* mRNA levels were increased in the *ccr4Δ* mutant, only Clb2, Clb4, and Clb6 protein levels showed a slight increase in the *ccr4Δ* mutant. Since we have previously shown that Ccr4 plays more important role in the regulation of gene expression in the stationary phase than in the log phase [11], elevated mRNA levels may not be important in the log phase but may be more important in the stationary phase. Since it is reported that translation efficiency is coupled with mRNA stability [45], the translation efficiency of *CLB* mRNAs may be involved in both mRNA and protein levels. Indeed, codon adaptation index of Clb proteins is relatively low: Clb1 0.14, Clb2, 0.12, Clb3 0.13, Clb4 0.13, Clb5 0.13, Clb6 0.17, Pgk1 0.81. Inefficient translation would account for the observation that increased mRNA levels do not confer the increase in the Clb protein levels. Recent study shows that the Ccr4-Not complex monitors the translating ribosome for codon optimality and links the decoding efficiency with mRNA stability [46]. Thus, Ccr4 may be directly involved in not only mRNA degradation but also translation efficiency.

### Both coding sequence and 3’ UTR of *CLB6* mRNA are involved in gene expression

We investigated the role of the coding region of *CLBs* in mRNA expression using the *CLBx-HA-ADH1 3’ UTR* plasmid and the *CLBx-HA-CLBx 3’ UTR* plasmid. Since all the *CLB1-6-HA-ADH1 3’ UTR* mRNA levels were increased in the *ccr4Δ* mutant compared to those in wild-type cells, the coding region of *CLBs* seems to have a role in mRNA expression. We suspected that a certain sequence within the coding region is responsible for the destabilization of the *CLB* mRNAs. Then we examined the region responsible for mRNA destabilization within *CLB6* coding sequence. We found that the *CLB6* coding sequences have three regions, 76–150 (the regions of D4 and D5), 201–278 (the regions of D9 and D10), and 201–278 (the region of D14) (Fig 7). Of these regions, 76–150 (the regions of D4 and D5) are different from the regions that encode the cyclin domain. The regions 201–278 (the regions of D9 and D10) and 201–278 (the region of D14) are the regions that encode the cyclin domain. The region encoding the cyclin domain may be involved in degradation at the mRNA level as well as at the protein level.

From the comparison of the results of the *CLBx-HA-CLBx 3’ UTR* plasmids and the *CLBx-HA-ADH1 3’ UTR* plasmids (Figs 7 and 8, Table 3), we found that the 3’ UTRs of *CLB5* and *CLB6* may have a role in mRNA level. This was further confirmed with the experiments using the *MCM2 promoter-GFP-CLBx 3’ UTR* plasmids. Since the *CLB6* 3’ UTR has the strongest effect, we examined the region responsible for mRNA destabilization within *CLB6* 3’ UTR. We found that the *CLB6* 3’ UTR has a region 37–83 (the regions of D3 and D4) (Fig 12). The results of D3 and D4 showed that the mRNA level did not rise so much, but the protein level rose significantly (Fig 12). Therefore, *CLB6* 3’ UTR controls both mRNA stability and translation efficiency. Our results suggest that the presence of sequences that control the amount of mRNA in both the coding region and the 3’ UTR determines the total amount of mRNA.

### Whi3, but not Puf proteins, is involved in the expression of *CLB* mRNA

Puf family RNA-binding proteins are known to recruit the Ccr4-Not deadenylase complex to the mRNAs promote decay [9,39,40]. However, our results using the *MCM2 promoter-GFP-CLBx 3’ UTR* plasmids suggested that Puf1-6 proteins are not involved in the Ccr4-dependent mRNA destabilization of *CLB6*. We found that Whi3 has a role in mRNA destabilization of *CLB* mRNAs. Whi3 is identified to have a role in stress-dependent RNA processing on various stress conditions [47] and a post-transcriptional control regulator of genes involved in cell division [48]. Whi3 is also known to bind to the *CLN3* mRNA and can cause sterility in old yeast cells [49]. Our results show that there was a significant increase in endogenous *CLB* mRNA with the deletion of this protein. Whi3 is known to have a protein-mRNA interaction with *CLB6* and this is a possible candidate as a regulatory-binding protein which interacts with Ccr4.

In this study, we have demonstrated the role of Ccr4 on the regulation of *CLB* expression. We have shown that this protein is a rate-limiting step on post-transcriptional regulation. Of all the *CLB* genes, it was *CLB6* which showed a potential to be degraded by Ccr4 by recognizing both its coding region and 3’ UTR. For further study, we plan to detect how exactly Ccr4 stabilizes the initiation of DNA synthesis stage, specifically the activation of Cdc28 via *CLB6*.

## Supporting information

S1 Fig Expression of Clb1-HA, Clb2-HA, Clb3-HA, Clb4-HA, Clb5-HA, and CLb6-HA proteins in wild-type and *ccr4*Δ mutant cells harboring the *CLBx-HA-CLBx 3’ UTR* plasmid. (TIFF)

S2 Fig Expression of Clb1-HA, Clb2-HA, Clb3-HA, Clb4-HA, Clb5-HA, and CLb6-HA proteins in wild-type and *ccr4*Δ mutant cells harboring the *CLBx-HA-ADH1 3’ UTR* plasmid. (TIFF)

S3 Fig Expression of GFP protein in wild-type strain harboring the *MCM2 promoter-GFP-CLBx 3’ UTR* plasmids. (TIFF)

S4 Fig Expression of GFP protein in wild-type and *ccr4Δ* mutant cells harboring the *MCM2 promoter-GFP-CLBx 3’ UTR* plasmids. (TIFF)

S5 Fig Deletion analysis of the *CLB6* coding sequence using the *CLB6-HA-CLB6 3’ UTR* plasmid. Protein levels were quantified by preparing cell extracts collected at log phase (4 H) for immunoblotting with anti-HA and anti-Pgk1 antibodies where Pgk1 was used as the loading control.
(TIFF)

S6 Fig Deletion analysis of the *CLB6* 3’ UTR using the *MCM2 promoter-GFP-CLB6 3’-UTR* plasmid. Protein levels were quantified by preparing cell extracts collected at log phase (4 H) for immunoblotting with anti-GFP and anti-Pgk1 antibodies where Pgk1 was used as the loading control.
(TIFF)

S1 Table Strains used in this study. (DOCX)

S2 Table Plasmids used in this study. (DOCX)

S3 Table Primers used for cloning of CLBx 3’ UTR. (DOCX)

S4 Table Primers used for constructing CLBx-HA-ADH1 3’ UTR plasmid. (DOCX)

S5 Table Primers used for constructing CLBx-HA-CLBx 3’ UTR plasmid. (DOCX)

S6 Table Primers used for the deletion sequences of *CLB6* coding region of 50 amino acids each. (DOCX)

S7 Table Primers used for the deletion sequences of *CLB6* 3’ UTR of 30 bases each. (DOCX)

S8 Table Primers for RT-PCR used in this study. (DOCX)
