# Supplementary figures and images for: Regulation of CLB6 expression by the cytoplasmic deadenylase Ccr4 through its coding and 3’ UTR regions
Source: PLoS One. 2022 May 6;17(5):e0268283. doi: 10.1371/journal.pone.0268283 (PMC9075657; doi:10.1371/journal.pone.0268283)

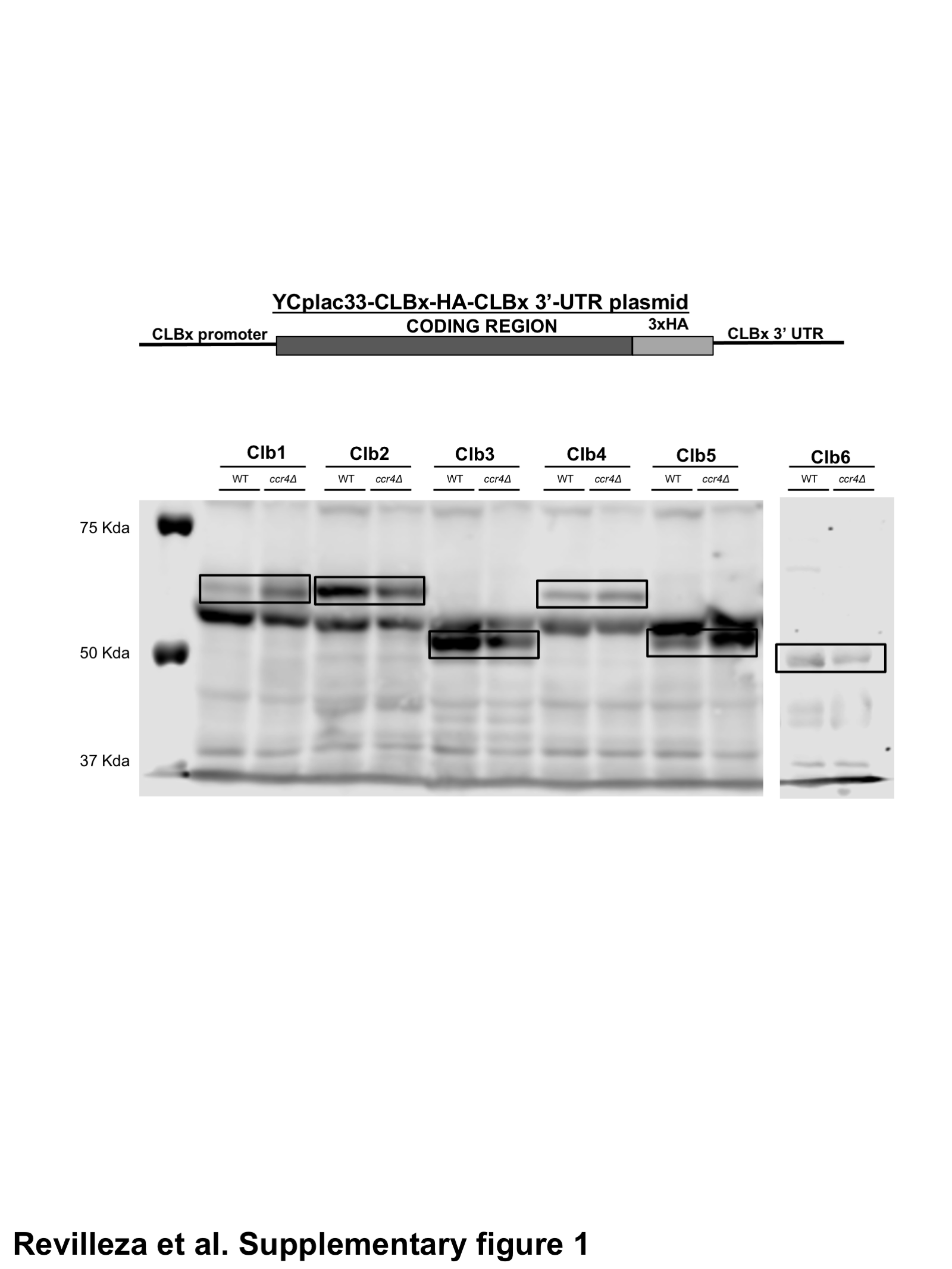

Supplement: S1 Fig — (TIFF) [file pone.0268283.s001.tiff]

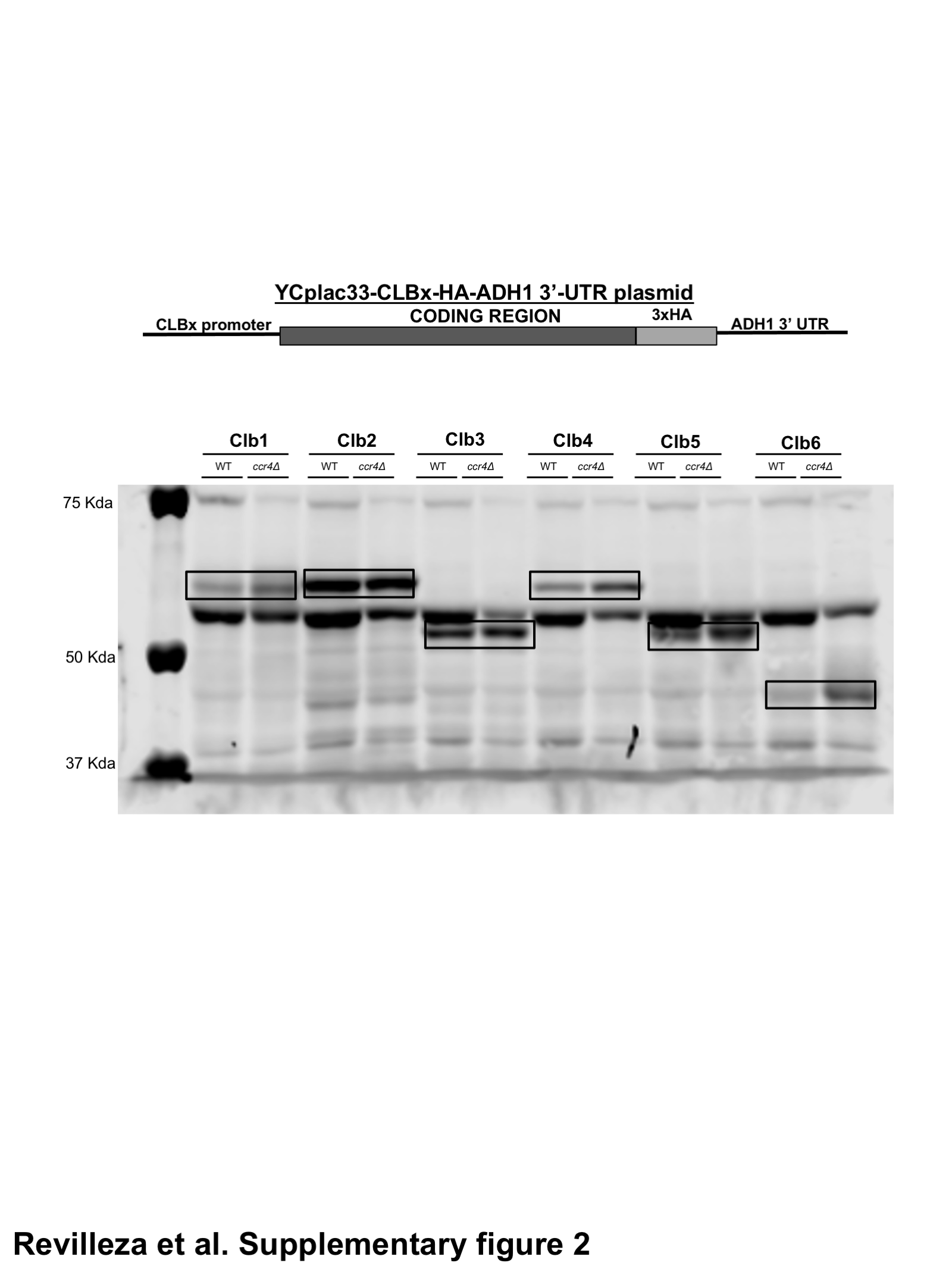

Supplement: S2 Fig — (TIFF) [file pone.0268283.s002.tiff]

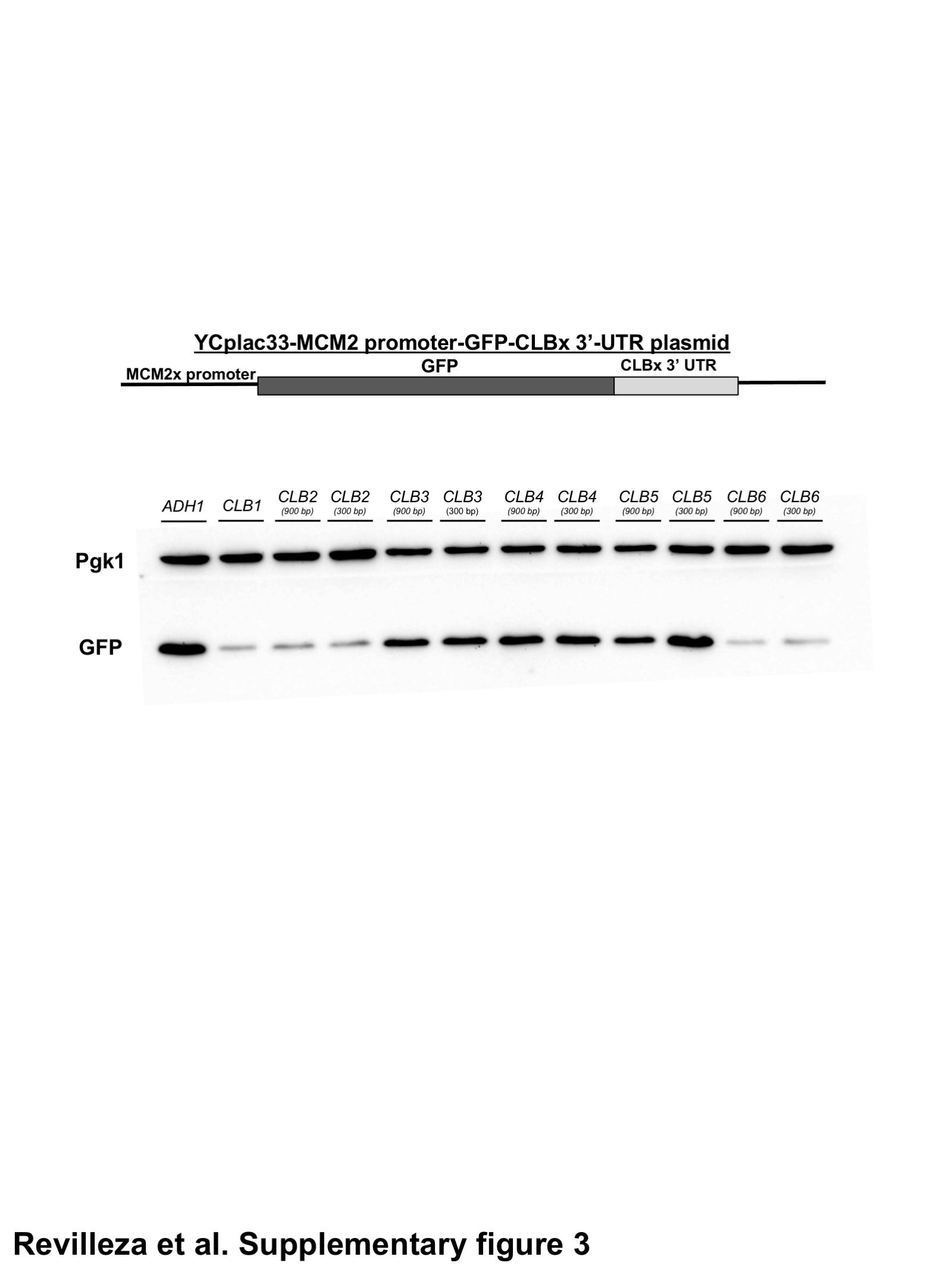

Supplement: S3 Fig — (TIFF) [file pone.0268283.s003.tiff]

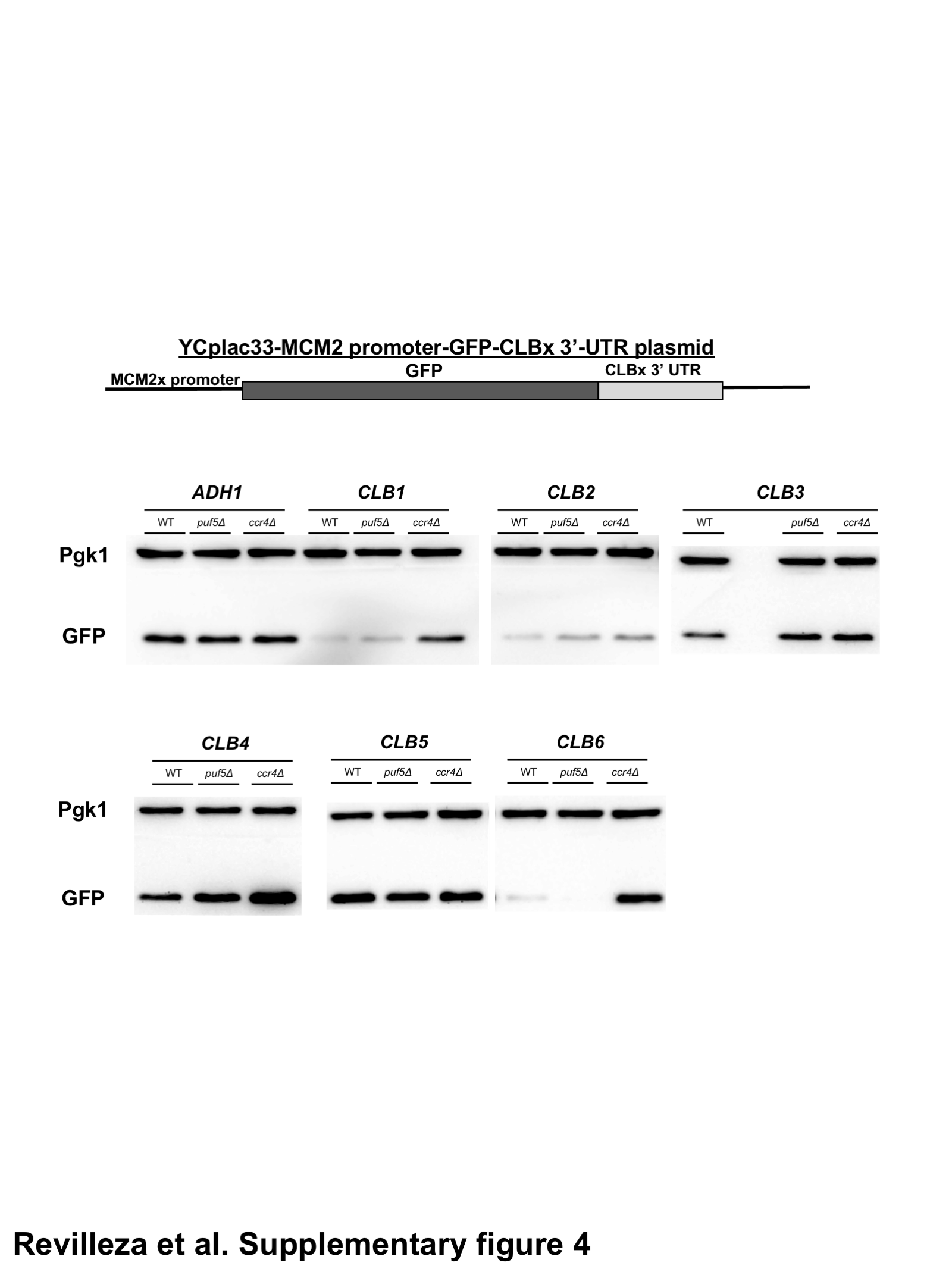

Supplement: S4 Fig — (TIFF) [file pone.0268283.s004.tiff]

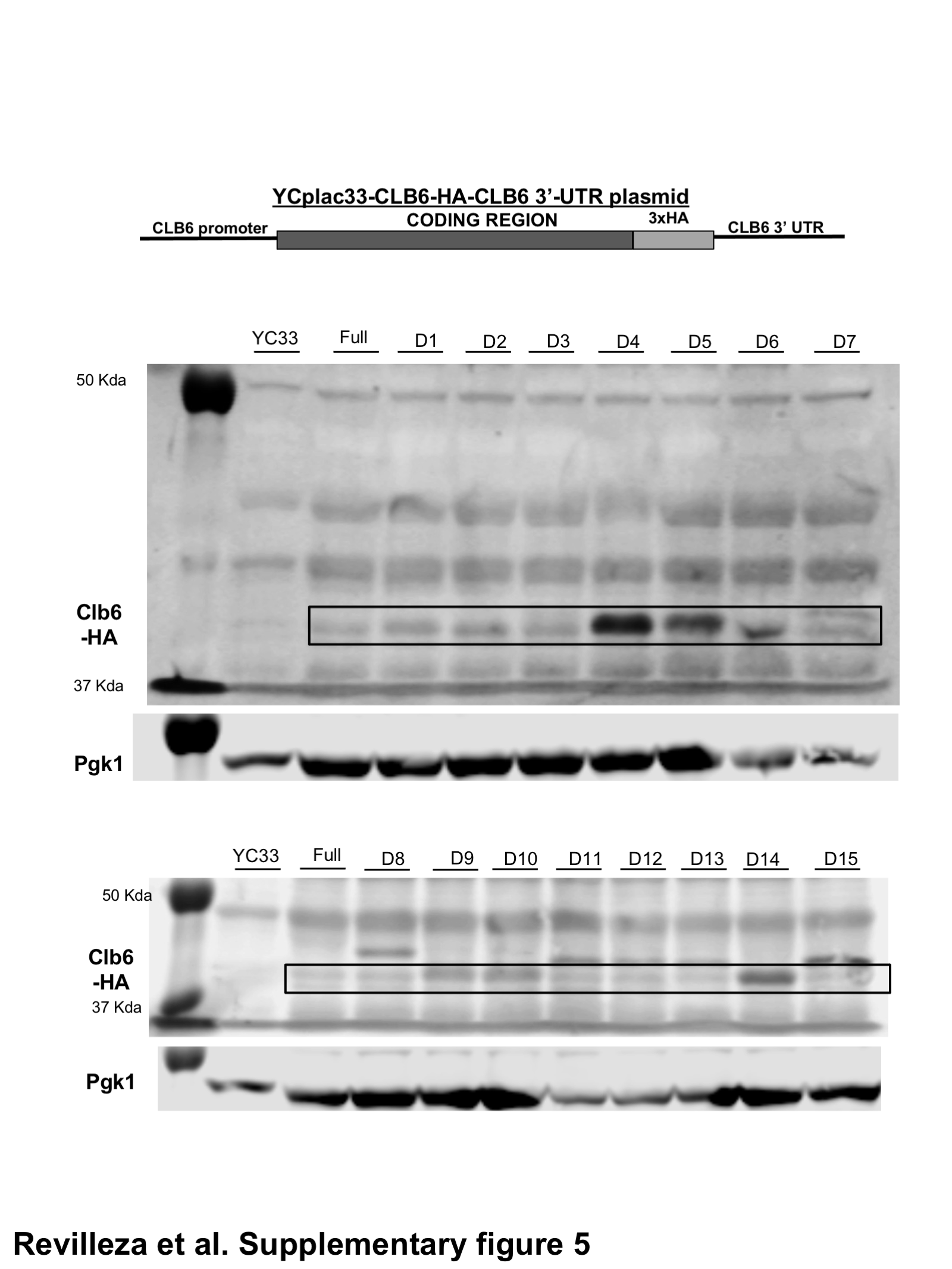

Supplement: S5 Fig — Protein levels were quantified by preparing cell extracts collected at log phase (4 H) for immunoblotting with anti-HA and anti-Pgk1 antibodies where Pgk1 was used as the loading control. (TIFF) [file pone.0268283.s005.tiff]

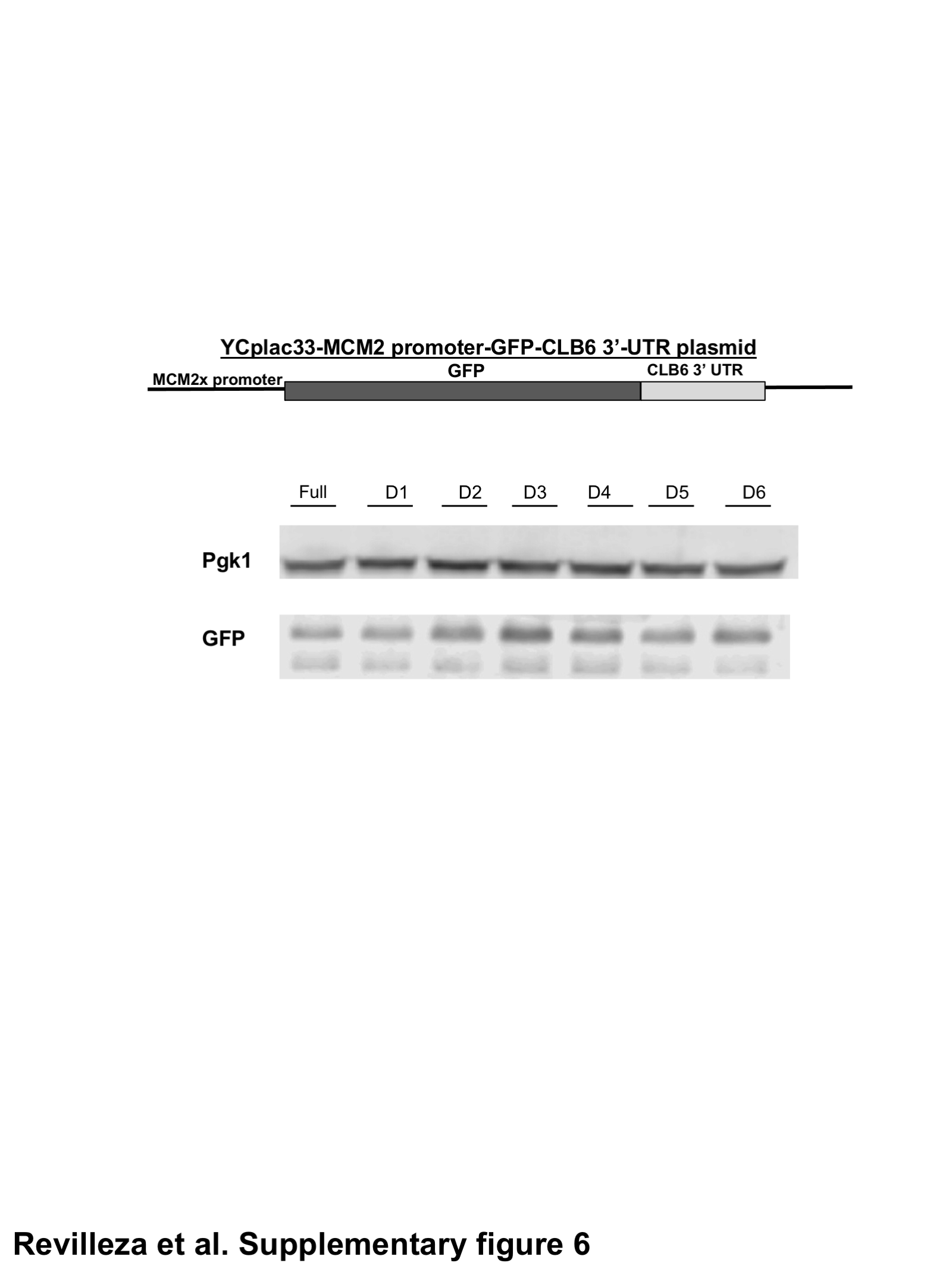

Supplement: S6 Fig — Protein levels were quantified by preparing cell extracts collected at log phase (4 H) for immunoblotting with anti-GFP and anti-Pgk1 antibodies where Pgk1 was used as the loading control. (TIFF) [file pone.0268283.s006.tiff]
