# Supplementary material for: Regulation of CLB6 expression by the cytoplasmic deadenylase Ccr4 through its coding and 3’ UTR regions
Source: PLoS One. 2022 May 6;17(5):e0268283. doi: 10.1371/journal.pone.0268283 (PMC9075657; doi:10.1371/journal.pone.0268283)
Supplement: S1 Table — (DOCX) [file pone.0268283.s007.docx]

**S1 Table. Strains used in this study**

| Strain | Genotype | Source |
| --- | --- | --- |
| 10B | *MATα ade2 trp1 can1 leu2 his3 ura3 GAL psi+ HOp-ADE2-HO 3' UTR* | 31 |
| 10BD | *MATa/MATα ade2/ade2 trp1/trp1 can1/can1 leu2/leu2 his3/his3 ura3/ura3* | 31 |
| 10BD-c4k1m1 | *MATa/MATα ade2/ade2 trp1/trp1 can1/can1 leu2/leu2 his3/his3 ura3/ura3 CCR4/ccr4Δ::CgLEU2 KHD1/khd1Δ::CgTRP1 MIH1/mih1Δ::CgHIS3* | This study |
| 10BD-c4k1s1 | *MATa/MATα ade2/ade2 trp1/trp1 can1/can1 leu2/leu2 his3/his3 ura3/ura3 CCR4/ccr4Δ::CgLEU2 KHD1/khd1Δ::CgTRP1 SWE1/swe1Δ::CgHIS3* | This study |
| *ccr4∆* | *MATa ade2 trp1 can1 leu2 his3 ura3 ccr4Δ::CgLEU2* | 34 |
| *pop2∆* | *MATa ade2 trp1 can1 leu2 his3 ura3 pop2Δ::CgLEU2* | 34 |
| *not1* | *MATa trp1 leu2 ura3 gcn4∆ cdc39-2* | 51 |
| *not2* | *MATa trp1 leu2 ura3 gcn4∆ cdc36* | 51 |
| *not4∆* | *MATa ade2 trp1 can1 leu2 his3 ura3 not4Δ::CgLEU2* | This study |
| *bar1∆* | *MATa ade2 trp1 can1 leu2 his3 ura3 bar1Δ::CgHIS3* | This study |
| *bar1∆ ccr4∆* | *MATa ade2 trp1 can1 leu2 his3 ura3 bar1Δ::CgHIS3 ccr4Δ::CgLEU2* | This study |
| *puf1∆* | *MATa ade2 trp1 can1 leu2 his3 ura3 puf1Δ::CgLEU2* | This study |
| *puf2∆* | *MATa ade2 trp1 can1 leu2 his3 ura3 puf2Δ::CgLEU2* | This study |
| *puf3∆* | *MATa ade2 trp1 can1 leu2 his3 ura3 puf3Δ::CgLEU2* | This study |
| *puf4∆* | *MATa ade2 trp1 can1 leu2 his3 ura3 puf4Δ::CgLEU2* | This study |
| *puf5∆* | *MATa ade2 trp1 can1 leu2 his3 ura3 puf5Δ::CgLEU2* | This study |
| *whi3Δ* | *MATa ade2 trp1 can1 leu2 his3 ura3 whi3Δ::CgLEU2* | This study |
| *caf20Δ* | *MATa ade2 trp1 can1 leu2 his3 ura3 caf20Δ::CgTRP1* | This study |
| *eap1Δ* | *MATa ade2 trp1 can1 leu2 his3 ura3 eap1Δ::CgLEU2* | This study |
| *caf20Δ eap1Δ* | *MATa ade2 trp1 can1 leu2 his3 ura3 caf20Δ::CgTRP1 eap1Δ::CgLEU2* | This study |

51. Collart MA, Struhl K. NOT1(CDC39), NOT2(CDC36), NOT3, and NOT4 encode a global-negative regulator of transcription that differentially affects TATA-element utilization. Genes Dev. 1994 Mar 1;8(5):525-37. doi: 10.1101/gad.8.5.525.
