# Supplementary material for: Regulation of CLB6 expression by the cytoplasmic deadenylase Ccr4 through its coding and 3’ UTR regions
Source: PLoS One. 2022 May 6;17(5):e0268283. doi: 10.1371/journal.pone.0268283 (PMC9075657; doi:10.1371/journal.pone.0268283)
Supplement: S2 Table — (DOCX) [file pone.0268283.s008.docx]

**S2 Table. Plasmids used in this study**

| Plasmids | Relevant markers | Reference |
| --- | --- | --- |
| YCplac33 | *URA3, CEN-ARS* | 52 |
| YCplac33- CLB1-HA-CLB1 3’-UTR | *URA3, CEN-ARS, CLB1-3HA* | This study |
| YCplac33- CLB2-HA-CLB2 3’-UTR | *URA3, CEN-ARS, CLB2-3HA* | This study |
| YCplac33- CLB3-HA-CLB3 3’-UTR | *URA3, CEN-ARS, CLB3-3HA* | This study |
| YCplac33- CLB4-HA-CLB4 3’-UTR | *URA3, CEN-ARS, CLB4-3HA* | This study |
| YCplac33- CLB5-HA-CLB5 3’-UTR | *URA3, CEN-ARS, CLB5-3HA* | This study |
| YCplac33- CLB6-HA-CLB1 3’-UTR | *URA3, CEN-ARS, CLB6-3HA* | This study |
| YCplac33- CLB1-HA-ADH1 3’-UTR | *URA3, CEN-ARS, CLB1-3HA* | This study |
| YCplac33- CLB2-HA-ADH1 3’-UTR | *URA3, CEN-ARS, CLB2-3HA* | This study |
| YCplac33- CLB3-HA-ADH1 3’-UTR | *URA3, CEN-ARS, CLB3-3HA* | This study |
| YCplac33- CLB4-HA-ADH1 3’-UTR | *URA3, CEN-ARS, CLB4-3HA* | This study |
| YCplac33- CLB5-HA-ADH1 3’-UTR | *URA3, CEN-ARS, CLB5-3HA* | This study |
| YCplac33- CLB6-HA-ADH1 3’-UTR | *URA3, CEN-ARS, CLB6-3HA* | This study |
| YCplac33-MCM2-GFP-ADH1 3’-UTR | *URA3, CEN-ARS, GFP* | This study |
| YCplac33- MCM2-GFP-CLB1 3’-UTR | *URA3, CEN-ARS, GFP* | This study |
| YCplac33- MCM2-GFP-CLB2 3’-UTR | *URA3, CEN-ARS, GFP* | This study |
| YCplac33- MCM2-GFP-CLB3 3’-UTR | *URA3, CEN-ARS, GFP* | This study |
| YCplac33- MCM2-GFP-CLB4 3’-UTR | *URA3, CEN-ARS, GFP* | This study |
| YCplac33- MCM2-GFP-CLB5 3’-UTR^,^ | *URA3, CEN-ARS, GFP* | This study |
| YCplac33- MCM2-GFP-CLB6 3’-UTR | *URA3, CEN-ARS, GFP* | This study |
| pCgLEU2 | *C. glabrata LEU2* in pUC19 | 25 |
| pCgHIS3 | *C. glabrata HIS3* in pUC19 | 25 |
| pCgTRP1 | *C. glabrata TRP1* in pUC19 | 25 |
| pFA6a-3HA-kanMX6 | *3HA-ADH1* terminator, kanamycin resistance cassette | 23 |

52. Gietz R D, Sugino A. New yeast-Escherichia coli shuttle vectors constructed with in vitro mutagenized yeast genes lacking six-base pair restriction sites. Gene. 1988 Dec 30;74(2):527-34. doi: 10.1016/0378-1119(88)90185-0.
