# Supplementary material for: Regulation of CLB6 expression by the cytoplasmic deadenylase Ccr4 through its coding and 3’ UTR regions
Source: PLoS One. 2022 May 6;17(5):e0268283. doi: 10.1371/journal.pone.0268283 (PMC9075657; doi:10.1371/journal.pone.0268283)
Supplement: S3 Table — (DOCX) [file pone.0268283.s009.docx]

**S3 Table. Primers used for cloning of CLBx 3’-UTR**

| 3’-UTR | Forward primer* | Reverse primer** |
| --- | --- | --- |
| *CLB1* | AGGTGCTAGCCTACACAGAAAACC | ATCAAAATTATAGCCAGCTAACCC |
| *CLB2* | GAAGAGGGGCAGATGCTTAAAATA | TTGTCTACCCTCGCTACATG |
| *CLB3* | AAAGCCTCAGCTCGAGACATGGCA | CTTTATCCCGCAAACCTTCC |
| *CLB4* | CTTACTCATCTTAACACCATTTGC | CTTCTACTGCGAATGCCGCTTAC |
| *CLB5* | ATTAGTACTAGTAATGCTCATGAA | TGTAGCGAGGGTAGACAAAG |
| *CLB6* | AACATAGATCAAAATTATAGCCAG | TGCATGAGTGAAGGTGCTAG |

*+ GAACTATACAAATAGGGCGCGCC – reverse complement of GFP TER region

**+ TTGTAAAACGACGGCCAGTGAATTC – ECOR1 site of YCplac33
