# Supplementary material for: Regulation of CLB6 expression by the cytoplasmic deadenylase Ccr4 through its coding and 3’ UTR regions
Source: PLoS One. 2022 May 6;17(5):e0268283. doi: 10.1371/journal.pone.0268283 (PMC9075657; doi:10.1371/journal.pone.0268283)
Supplement: S4 Table — (DOCX) [file pone.0268283.s010.docx]

**S4 Table. Primers used for constructing CLBx-3HA-ADH1 3’-UTR plasmid**

| 3’-UTR | 45-bp upstream stop codon* | 45-bp downstream stop codon (complementary)** |
| --- | --- | --- |
| *CLB2* | TTAAAGGTTAGAAAAAACGGCTATGATATAATGACCTTGCATGAA | TTTATCGATTATCGTTTTAGATATTTTAAGCATCTGCCCCTCTTC |
| *CLB3* | GCGAAGTGGATAGCATTAGCTGAACACAGAGTAGAAAGATCTAAC | GAATCCTTTTTCCTTTGTTGATGCCATGTCTCGAGCTGAGGCTTT |
| *CLB4* | CGCTGTTCTCAAATTGTAGAAGAATGGATTGTTTCGACAGAAGCC | CCTTCCGAAACCAAAACTGAAGCAAATGGTGTTAAGATGAGTAAG |
| *CLB5* | ACATCCGAAATGCATAGCAACTTTCAAAATCTATTTAATCTTAAG | AAAATGTAAAGAGTATGCGAATTCATGAGCATTACTAGTACTAAT |
| *CLB6* | GGCATGGTTTATTTCAAGGTTTTTGACTGGTGTAAACAAAAACGT | ATTTAAGATGCAGGGGGTTAGCTGGCTATAATTTTGATCTATGTT |
