# Supplementary material for: Regulation of CLB6 expression by the cytoplasmic deadenylase Ccr4 through its coding and 3’ UTR regions
Source: PLoS One. 2022 May 6;17(5):e0268283. doi: 10.1371/journal.pone.0268283 (PMC9075657; doi:10.1371/journal.pone.0268283)
Supplement: S5 Table — (DOCX) [file pone.0268283.s011.docx]

**S5 Table. Primers used for constructing CLBx-3HA-CLBx 3’-UTR plasmid**

|  | Forward primer* | Reverse primer (complementary)** |
| --- | --- | --- |
| *CLB2* | ACCCTGTTCTTGACCGTCTACTAAGG | GATATTTTAAGCATCTGCCCCTCTTC |
| *CLB3* | TATGACGATTCACTGGGTATCAGCG | GCCATGTCTCGAGCTGAGGCTTT |
| *CLB4* | TCGGGAACAGTACTCATTCCGACAG | CTGAAGCAAATGGTGTTAAGATGAGTAAG |
| *CLB5* | TTTTATGTCCCCACGTTACACCGCATG | GCGAATTCATGAGCATTACTAGTACTAAT |
| *CLB6* | CTGTTGGTCCTTAGTTCGATCCTGAG | GGGGGTTAGCTGGCTATAATTTTGATCTATGTT |
