# Supplementary material for: Regulation of CLB6 expression by the cytoplasmic deadenylase Ccr4 through its coding and 3’ UTR regions
Source: PLoS One. 2022 May 6;17(5):e0268283. doi: 10.1371/journal.pone.0268283 (PMC9075657; doi:10.1371/journal.pone.0268283)
Supplement: S6 Table — (DOCX) [file pone.0268283.s012.docx]

**S6 Table. Primers used for the deletion sequences of *CLB6* coding region of 50 amino acids each**

| Deletion | Amino acid deletion | Deleted Sequence | Forward primer | Reverse primer |
| --- | --- | --- | --- | --- |
| 1 | 2-50 | AATTGTATCCCTAGTCCAATTAGTGAAAGGAAAATCCAAATTAATAATGAAGATTGTATAGGCAAGGAAAATGCTTTCCATACCATTCCAAGAGAAAGTTCAATTAACTTGACACCTCACTCTACGAATGAAAAAAAAGTTCTATCC | CCCTCCTTTTAAATTTTTAAAATGGAGGTAAACAGTAACAAAATCGAT | ATCGATTTTGTTACTGTTTACCTCCATTTTAAAAATTTAAAAGGAGGG |
| 2 | 25-75 | AATGCTTTCCATACCATTCCAAGAGAAAGTTCAATTAACTTGACACCTCACTCTACGAATGAAAAAAAAGTTCTATCCGAGGTAAACAGTAACAAAATCGATTCTTTACAACTCCCTCGAGGTAAATTGCAAAGAGATTCCACCCATTTGGAA | AATGAAGATTGTATAGGCAAGGAAAAAACAAGAAAGAGACAATTATCA | TGATAATTGTCTCTTTCTTGTTTTTTCCTTGCCTATACAATCTTCATT |
| 3 | 51-100 | GAGGTAAACAGTAACAAAATCGATTCTTTACAACTCCCTCGAGGTAAATTGCAAAGAGATTCCACCCATTTGGAAAAAACAAGAAAGAGACAATTATCAAATGATTCAACCGATCCTATAGAGCCAAAAACTGTGAAAAAAATAAAATGT | ACGAATGAAAAAAAAGTTCTATCCCATCAATGGAAAAATTTGGATTCG | CGAATCCAAATTTTTCCATTGATGGGATAGAACTTTTTTTTCATTCGT |
| 4 | 76-125 | AAAACAAGAAAGAGACAATTATCAAATGATTCAACCGATCCTATAGAGCCAAAAACTGTGAAAAAAATAAAATGTCATCAATGGAAAAATTTGGATTCGATAGAGATGGATGATCCATTCATGGTAGCAGAATATACAGATTCTATATTT | CAAAGAGATTCCACCCATTTGGAATCTCATCTTTACGAGAAGGAAATC | GATTTCCTTCTCGTAAAGATGAGATTCCAAATGGGTGGAATCTCTTTG |
| 5 | 101-150 | CATCAATGGAAAAATTTGGATTCGATAGAGATGGATGATCCATTCATGGTAGCAGAATATACAGATTCTATATTTTCTCATCTTTACGAGAAGGAAATCCAAATGCTACCGACACATAACTATTTAATGGACACGCAATCTCCCTATCAT | AAAACTGTGAAAAAAATAAAATGTTTGAAAAGCTCCATGAGGGCGCTG | CAGCGCCCTCATGGAGCTTTTCAAACATTTTATTTTTTTCACAGTTTT |
| 6 | 126-176 | TCTCATCTTTACGAGAAGGAAATCCAAATGCTACCGACACATAACTATTTAATGGACACGCAATCTCCCTATCATTTGAAAAGCTCCATGAGGGCGCTGTTAATTGACTGGTTGGTTGAAGTTCATGAGAAATTTCACTGCTTACCTGAAACA | GCAGAATATACAGATTCTATATTTTTATTTTTAGCGATCAACCTGCTA | TAGCAGGTTGATCGCTAAAAATAAAAATATAGAATCTGTATATTCTGC |
| 7 | 151-200 | TTGAAAAGCTCCATGAGGGCGCTGTTAATTGACTGGTTGGTTGAAGTTCATGAGAAATTTCACTGCTTACCTGAAACATTATTTTTAGCGATCAACCTGCTAGATCGATTTTTATCACAAAATGTTGTTAAATTGAATAAATTACAACTA | ATGGACACGCAATCTCCCTATCATCTATGCATCACTTGCCTGTTCATT | AATGAACAGGCAAGTGATGCATAGATGATAGGGAGATTGCGTGTCCAT |
| 8 | 177-227 | TTATTTTTAGCGATCAACCTGCTAGATCGATTTTTATCACAAAATGTTGTTAAATTGAATAAATTACAACTACTATGCATCACTTGCCTGTTCATTGCCTGTAAATTTGAAGAAGTTAAATTACCAAAAATAACAAATTTTGCGTACGTGACT | AAATTTCACTGCTTACCTGAAACAGACGGTGCTGCCACTGTCGAAGGA | TCCTTCGACAGTGGCAGCACCGTCTGTTTCAGGTAAGCAGTGAAATTT |
| 9 | 201-250 | CTATGCATCACTTGCCTGTTCATTGCCTGTAAATTTGAAGAAGTTAAATTACCAAAAATAACAAATTTTGCGTACGTGACTGACGGTGCTGCCACTGTCGAAGGAATTAGGAAGGCTGAGCTATTCGTTCTAAGTTCTTTAGGTTATAAC | GTTAAATTGAATAAATTACAACTAATATCTCTACCAAACCCCCTAAAT | ATTTAGGGGGTTTGGTAGAGATATTAGTTGTAATTTATTCAATTTAAC |
| 10 | 228-278 | GACGGTGCTGCCACTGTCGAAGGAATTAGGAAGGCTGAGCTATTCGTTCTAAGTTCTTTAGGTTATAACATATCTCTACCAAACCCCCTAAATTTCATTAGGAGAATTTCAAAAGCTGACAATTACTGCATTGAAACAAGGAACATGGCCAAA | ATAACAAATTTTGCGTACGTGACTTTCATCATGGAATATTCGATTTGT | ACAAATCGAATATTCCATGATGAAAGTCACGTACGCAAAATTTGTTAT |
| 11 | 251-300 | ATATCTCTACCAAACCCCCTAAATTTCATTAGGAGAATTTCAAAAGCTGACAATTACTGCATTGAAACAAGGAACATGGCCAAATTCATCATGGAATATTCGATTTGTTGTAATAAATTCATCCACCTGAAGCCATCATATTTAGCTGCA | GTTCTAAGTTCTTTAGGTTATAACATGTCCATGTATATCGCCAGAAAA | TTTTCTGGCGATATACATGGACATGTTATAACCTAAAGAACTTAGAAC |
| 12 | 279-329 | TTCATCATGGAATATTCGATTTGTTGTAATAAATTCATCCACCTGAAGCCATCATATTTAGCTGCAATGTCCATGTATATCGCCAGAAAAATTAAAAATGAAAATTCCAAATGGGATGAAACTTTTATTCATTATAGTGGCGGCATTGATATT | ATTGAAACAAGGAACATGGCCAAAGAATCAGATCCAGCGTTTAAGGAT | ATCCTTAAACGCTGGATCTGATTCTTTGGCCATGTTCCTTGTTTCAAT |
| 13 | 301-350 | ATGTCCATGTATATCGCCAGAAAAATTAAAAATGAAAATTCCAAATGGGATGAAACTTTTATTCATTATAGTGGCGGCATTGATATTGAATCAGATCCAGCGTTTAAGGATTTTATCAGTGAACTAGTTGAAGATATTGCTGTACCTGAT | CTGAAGCCATCATATTTAGCTGCAACGAATCTAGATTCCTTGAGACTT | AAGTCTCAAGGAATCTAGATTCGTTGCAGCTAAATATGATGGCTTCAG |
| 14 | 330-380 | GAATCAGATCCAGCGTTTAAGGATTTTATCAGTGAACTAGTTGAAGATATTGCTGTACCTGATACGAATCTAGATTCCTTGAGACTTAAATACAAAAAGCCTAAACATGGCATGGTTTATTTCAAGGTTTTTGACTGGTGTAAACAAAAACGT | CATTATAGTGGCGGCATTGATATTCGGATCCCCGGGTTAATTAACATCTTTTAC | GTAAAAGATGTTAATTAACCCGGGGATCCGAATATCAATGCCGCCACTATAATG |
| 15 | 351-381 | ACGAATCTAGATTCCTTGAGACTTAAATACAAAAAGCCTAAACATGGCATGGTTTATTTCAAGGTTTTTGACTGGTGTAAACAAAAACGT | GTTGAAGATATTGCTGTACCTGATCGGATCCCCGGGTTAATTAACATCTTTTAC | GTAAAAGATGTTAATTAACCCGGGGATCCGATCAGGTACAGCAATATCTTCAAC |
