# Supplementary material for: Regulation of CLB6 expression by the cytoplasmic deadenylase Ccr4 through its coding and 3’ UTR regions
Source: PLoS One. 2022 May 6;17(5):e0268283. doi: 10.1371/journal.pone.0268283 (PMC9075657; doi:10.1371/journal.pone.0268283)
Supplement: S7 Table — (DOCX) [file pone.0268283.s013.docx]

**S7 Table. Primers used for the deletion sequences of *CLB6* 3’-UTR of 30 bases each**

| Deletion | Amino acid deletion | Deleted Sequence | Forward primer | Reverse primer |
| --- | --- | --- | --- | --- |
| 1 | 2-12 | ATAGATCAAAATTATAGCCAGCTAACCCCCTGCATC | GAACTATACAAATAGGGCGCGCCAACATCTTAAATATCATCGATAGACAT | ATGTCTATCGATGATATTTAAGATGTTGGCGCGCCCTATTTGTATAGTTC |
| 2 | 7-17 | AGCCAGCTAACCCCCTGCATCTTAAATATCATC | CGCGCCAACATAGATCAAAATTATGATAGACATACTTCTTTATTTCTT | AAGAAATAAAGAAGTATGTCTATCATAATTTTGATCTATGTTGGCGCG |
| 3 | 13-23 | CTGCATCTTAAATATCATCGATAGACATACTTCTTTA | AATTATAGCCAGCTAACCCCCTGCTTTCTTTTATGTATTCAAAAAAAG | CTTTTTTTGAATACATAAAAGAAAGCAGGGGGTTAGCTGGCTATAATT |
| 4 | 18-28 | GATAGACATACTTCTTTATTTCTTTTATGTA | ACCCCCTGCATCTTAAATATCATCTTCAAAAAAAGAAAGAAAATGCAT | ATGCATTTTCTTTCTTTTTTTGAAGATGATATTTAAGATGCAGGGGGT |
| 5 | 24-34 | TTTCTTTTATGTATTCAAAAAAAGAAAGAAAAT | ATCATCGATAGACATACTTCTTTAGCATTATTCCTCACTTTAAAGATA | TATCTTTAAAGTGAGGAATAATGCTAAAGAAGTATGTCTATCGATGAT |
| 6 | 29-39 | TTCAAAAAAAGAAAGAAAATGCATTATTCCTCACT | ATACTTCTTTATTTCTTTTATGTATTAAAGATAATTTTCCAATTATGG | CCATAATTGGAAAATTATCTTTAATACATAAAAGAAATAAAGAAGTAT |
