# Supplementary material for: Regulation of CLB6 expression by the cytoplasmic deadenylase Ccr4 through its coding and 3’ UTR regions
Source: PLoS One. 2022 May 6;17(5):e0268283. doi: 10.1371/journal.pone.0268283 (PMC9075657; doi:10.1371/journal.pone.0268283)
Supplement: S8 Table — (DOCX) [file pone.0268283.s014.docx]

**S8 Table. Primers for RT-PCR used in this study**

|  | Forward primer | Reverse primer |
| --- | --- | --- |
| *ACT1* | TGCCGAAAGAATGCAAAAGG | TCTGGAGGAGCAATGATCTTGA |
| *SCR1* | AACCGTCTTTCCTCCGTCGTAA | CTACCTTGCCGCACCAGACA |
| *GFP* | CACTGGAGTTGTCCCAATTCTTG | TCCGTATGTTGCATCACCTTCA |
| *CLB1* | TTCCGAGCAAGAAAAGCAGC | TCGTACTCCTCCAGAACCTC |
| *CLB2* | GCCGATGACTTCACCTCCTC | CTGCTGCTTTTCTTGCTCGG |
| *CLB3* | GGAACAAGAGGAACCCGTTG | AATTCGGCAACCATGACCAC |
| *CLB4* | CAGCAGATTCAAGCCGATGA | AGCGTACTCCACAACCATCA |
| *CLB5* | ACGGCAGCAGAGCAAGAAGA | TCTAGGTCCTGCCAGCCTAC |
| *CLB6* | GCGATCAACCTGCTAGATCG | ACGAATAGCTCAGCCTTCCT |
| *RNR1* | CCATGGCACCAATGCCTACT | CACCGGATAAGACACGACGG |
| *SIC1* | GGCTTACGTCTCCTCAACGC | CGACCCAATGGTTCCTGCTC |
| *CLB1-3HA* | GAGCGGATCCCCGGGTTAAT | TAGTCCGGGACGTCATACGG |
| *CLB2-3HA* | CCTTGCATGAACGGATCCCC | CGTCATACGGATAGCCCGCA |
| *CLB3-3HA* | AACCGGATCCCCGGGTTAAT | TAGTCCGGGACGTCATACGG |
| *CLB4-3HA* | TGTTTCGACAGAAGCCCGGA | CGTCATACGGATAGCCCGCA |
| *CLB5-3HA* | AAGCGGATCCCCGGGTTAAT | TAGTCCGGGACGTCATACGG |
| *CLB6-3HA* | CGTCGGATCCCCGGGTTAAT | TAGTCCGGGACGTCATACGG |
